# Valence‐Engineering of CeO_2_ Redox Modulator Boosts the Oxygen Electrocatalysis Performance in Fe/Co Dual‐Atom Catalyst

**DOI:** 10.1002/advs.202516405

**Published:** 2025-12-20

**Authors:** Hengqi Liu, Jinzhen Huang, Shengyu Ma, Rui Xiong, Jiong Zhao, Qiang Fu, Hang Wei, Zhiguo Liu, Xianjie Wang, Tai Yao, Bo Song

**Affiliations:** ^1^ School of Physics Harbin Institute of Technology Harbin 150001 China; ^2^ PSI Center for Energy and Environmental Sciences Villigen PSI CH‐5232 Switzerland; ^3^ School of future technology Harbin Institute of Technology Harbin 150001 China; ^4^ Department of Applied Physics The Hong Kong Polytechnic University Kowloon Hong Kong 999077 China; ^5^ College of Chemistry and Chemical Engineering Inner Mongolia Engineering and Technology Research Center for Catalytic Conversion and Utilization of Carbon Resource Molecules Inner Mongolia University Hohhot 010021 China; ^6^ National Key Laboratory of Science and Technology on Advanced Composites in Special Environments Harbin Institute of Technology Harbin 150001 China; ^7^ Laboratory for Space Environment and Physical Sciences Harbin Institute of Technology Harbin Institute of Technology Harbin 150001 China; ^8^ National Key Laboratory of Laser Spatial Information Harbin Institute of Technology Harbin Institute of Technology Harbin 150001 China; ^9^ Frontier Research Center of Space Environment Interacting with Matter Harbin Institute of Technology Harbin 150001 China; ^10^ Zhengzhou Research Institute Harbin Institute of Technology Zhengzhou 450046 China

**Keywords:** dual‐atom catalysts, oxygen evolution reaction, oxygen reduction reaction, rechargeable zinc‐air batteries, valence engineering

## Abstract

Dual‐atom nitrogen‐doped carbon catalysts have garnered considerable interest as air electrodes for rechargeable zinc‐air batteries (ZABs), owing to their outstanding bifunctional oxygen electrocatalytic activity. Nonetheless, their practical implementation is hindered by durability issues during the oxygen reduction reaction (ORR), as the active sites are degraded by the oxidative by‐products. Furthermore, the sluggish electron transfer obstructs the oxidation state transitions of Co/Fe centers to limit the oxygen evolution reaction (OER) activity. Here, the valence engineering is performed in CeO_2_ redox modulator that is composed with Fe/Co dual‐atom catalyst (FeCo─N─C), to resolve the stability and activity issues. The valence‐engineered CeO_2_ with a fast Ce^4+^/Ce^3+^ redox process can efficiently scavenge the highly oxidative by‐products in ORR to improve the durability. In addition, the interaction of CeO_2_ with FeCo─N─C can accelerate electron transfer and lower the Co redox potential during the OER. The ZABs assembled with optimized FeCo─N─C/CeO_2_ as the air electrode display a maximum power density of 335 mWcm^−2^ without significant deterioration after ≈1000 h of continuous cycling. Therefore, compositing with valence‐engineered CeO_2_ provides a simple and effective solution to resolve the stability and activity issues of FeCo─N─C as an air electrode.

## Graphical abstract

Valence‐engineering of CeO_2_ redox modulator can remove the highly oxidative by‐products and accelerate the oxidation state transitions of Co/Fe centers in FeCo dual‐atom catalyst during oxygen reduction/evolution electrocatalysis. Therefore, the stability and activity issues are resolved, significantly enhancing the overall performance when used as an air electrode in zinc‐air batteries.

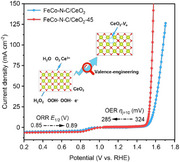

## 1 Introduction

With the increasing demand for renewable energy sources, rechargeable zinc‐air batteries (ZABs) have emerged as attractive alternatives for electrochemical energy storage devices due to their high energy density and environmental friendliness.^[^ ^1^, ^2^ ^]^ The total performance of ZABs is largely determined by the air electrode catalysts.^[^ ^3^, ^4^ ^]^ Dual‐atom catalysts have attracted considerable interest due to their exceptional atomic usage efficiency, distinctive electronic configurations, and multifunctional synergistic electrocatalytic characteristics.^[^ ^5^, ^6^ ^]^ In particular, the Fe‐N and Co‐O units are positioned near the peak of the ORR and OER volcano plots, respectively,^[^ ^7^, ^8^, ^9^ ^]^ therefore the integration of these two active centers into dual‐atom catalysts is highly promising to achieve the optimized oxygen electrocatalysis performance. However, like other single/dual‐atom catalysts, FeCo dual‐atom catalysts (FeCo‐N‐C) also suffer from poor durability during ORR, due to the degradation of the metal sites by corrosive byproducts (e.g., H_2_O_2_, HO_2_·, and ·OH).^[^ ^10^, ^11^ ^]^ To address this issue, significant efforts have been devoted to improving the graphitization degree of the carbon substrate^[^ ^12^ ^]^ and reducing the loading of metal precursors.^[^ ^13^ ^]^ Nevertheless, these passive shielding techniques usually diminish the accessibility of active sites. Alternatively, an active defense strategy is advocated—directly neutralizing highly oxidative species to prevent the catalytic deterioration at its origin,^[^ ^14^, ^15^ ^]^ to improve the durability of dual‐atom catalysts while maintaining their ORR activity.

Furthermore, the active sites for the OER are metal‐O units,^[^ ^16^ ^]^ necessitating the metal sites in dual‐atom configurations to engage in oxidation‐state transformation to form the OER intermediates. The ability to modify the electronic state is essential for regulating the adsorption and desorption of reaction intermediates, which directly influences the overall activity and efficiency of the reaction.^[^ ^17^, ^18^, ^19^ ^]^ However, single/dual‐atom catalysts generally exhibit poor valence variability as a result of their inherent structural characteristics.^[^ ^20^, ^21^ ^]^ To overcome this limitation, heteroatom doping^[^ ^22^ ^]^ or altering the geometry of the central atom coordination^[^ ^23^ ^]^ in dual‐atom structures has been successfully implemented. Nevertheless, the linear scaling relationships between the OER and ORR processes^[^ ^24^ ^]^ frequently result in the enhancement of OER activity at the expense of ORR performance. OER performance may be improved without sacrificing ORR activity if a modulator is added outside the dual‐atom catalyst structure to induce the electronic interactions surrounding the dual‐atom centers in response to voltage variations.

For instance, CeO_2_ is less active for OER itself, but can enhance the catalytic performance when combined with other transition metal compounds, due to its unique electronic modulation capabilities.^[^ ^25^, ^26^, ^27^ ^]^ Integration of CeO_2_ with dual‐atom catalysts to enhance OER performance is promising but underexplored. Moreover, enhancing the conductivity of CeO_2_ modulators enables more rapid and effective electron absorption at reaction sites, thus boosting reaction and decreasing energy losses.^[^ ^26^, ^28^ ^]^ The incorporation of mixed‐valence CeO_2_ through a valence‐engineering strategy can effectively enhance its conductivity, potentially achieving a compromise between electron absorption efficiency and conductivity. More importantly, since Ce^4+^ reacts with H_2_O_2_ and·OOH, while Ce^3+^ interacts with·OH, the interconversion between Ce^3+^ and Ce^4+^ in CeO_2_ can efficiently eliminate a variety of highly oxidative species during this cyclic process,^[^ ^29^ ^]^ thereby reducing their degradation of the metal‐N*_x_* sites during the ORR process.

Based on the above considerations, we have synthesized composite catalysts consisting of valence‐engineered CeO_2_ redox modulator and FeCo─N─C (denoted as FeCo─N─C/CeO_2_‐X, where X refers to the different soaking time in NaBH_4_ solution to engineer the Ce^4+^/Ce^3+^ ratio by reduction). Confirmed by fluorescence measurement and density functional theory (DFT) calculations, we prove that the valence‐engineered CeO_2_ can effectively scavenge the free radicals via redox process between Ce^3+^ and Ce^4+^. Additionally, CeO_2_ can modify the electron transfer process around FeCo atomic sites and regulate the redox process during the OER. Consequently, the optimized FeCo─N─C/CeO_2_‐45 catalyst exhibited exceptional oxygen electrocatalytic activity (*E*_1/2_ = 0.89 V for ORR and *η*_10_ = 285 mV for OER) and outstanding stability. When tested as the air electrode catalyst in a ZABs, the system attained a maximum power density of 335 mW cm^−2^ and sustained a high round‐trip efficiency after 1,000 h of continuous cycling. This study presents a simple and effective method to overcome the challenges faced by dual‐atom nitrogen‐doped carbon catalysts in ZABs.

## 2 Results and Discussion

### 2.1 Theoretical Predictions

**Figure 1:**
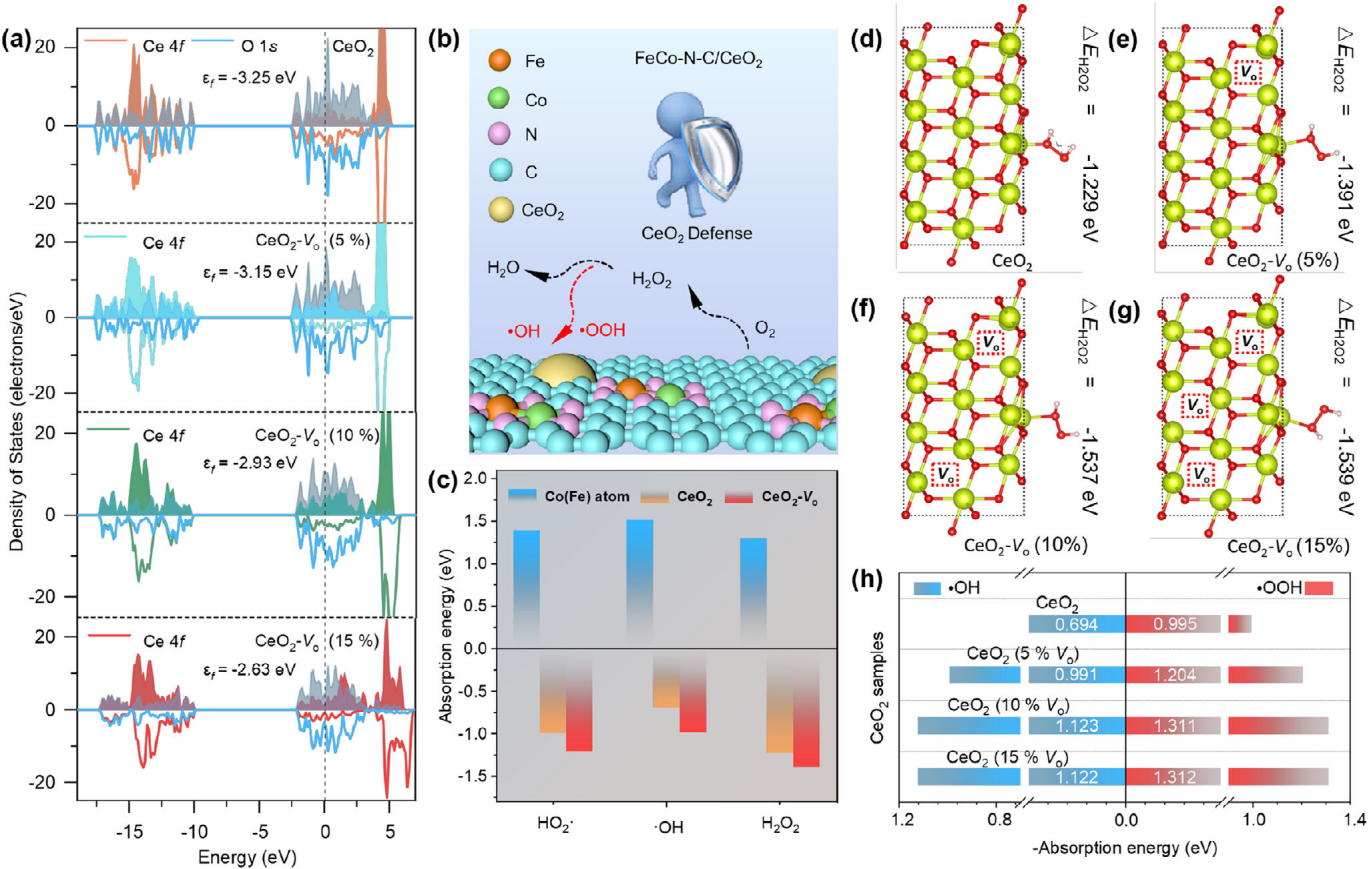
DFT verification of the effects of oxygen vacancy content in CeO_2_. a) PDOSs of the CeO_2_, CeO_2_‐*V*_O_ (5%), CeO_2_‐*V*_O_ (10%) and CeO_2_‐*V*_O_ (15%). b) Schematic illustration of CeO_2_ absorbing strongly oxidative byproducts on the FeCo─N─C support. c) Adsorption energies of H_2_O_2_ and related radicals on the surfaces of Co (Fe) atom, CeO_2,_ and CeO_2_‐*V*_O_. The models of d) CeO_2_, e) CeO_2_‐*V*_O_ (5%), f) CeO_2_‐*V*_O_ (10%), and g) CeO_2_‐*V*_O_ (15%) after H_2_O_2_ adsorption and their corresponding adsorption energies. h) Statistical results of the adsorption energies between CeO_2_ with varying *V*_O_ concentrations and ·OH/H_2_O_2_ species.

DFT calculations were first performed to investigate the scavenging capacity of mixed‐valence CeO_2_ toward highly oxidative by‐products. Cerium is mostly found in the +4‐oxidation state in pristine CeO_2_. To preserve charge neutrality, the introduction of oxygen vacancies causes Ce^4+^ to be partially reduced to Ce^3+^. Thus, the Ce^4+^/Ce^3+^ ratio is controlled by the concentration of oxygen vacancies, which in turn modifies the electronic structure and surface reactivity of CeO_2_. Based on this, the oxygen atoms in lattices were gradually removed to create faulty CeO_2_ models with oxygen vacancy concentrations of 5%, 10%, and 15% (Figure S1a–d, Supporting Information). To further elucidate the impact of oxygen vacancies (*V*_O_) on the adsorption behavior toward H_2_O_2_ and the related free radicals, the partial density of states (PDOS) was calculated (**Figure** **1**a). The center of the Ce 4*f* orbital moves upward from −3.25 eV (CeO_2_) to −2.63 eV (CeO_2_‐*V*_O_ (15%)) as the percentage of oxygen vacancies increases, enhancing the capacity of CeO_2_ to absorb the highly oxidative by‐products.

As shown in Figure 1b, reactive by‐products generated during the ORR by FeCo─N─C can attack Fe─N_x_ and Co─N_x_ active centers. Owing to its exceptional redox properties and reversible Ce^4+^/Ce^3+^ cycling capability, CeO_2_ with different *V*_O_ concentrations can adsorb and neutralize these by‐products. Therefore, the adsorption energies of H_2_O_2_, OH, and·OOH on the structures of FeCo─N─C (Fe sites and Co sites), CeO_2,_ and CeO_2_‐*V*_O_ were calculated to reveal their capabilities in capturing oxidative by‐products (Figure 1c). Among them, the CeO_2_‐*V*_O_ surface exhibited the lowest negative adsorption energies (i.e., the adsorption is exothermic) for H_2_O_2_,·OH, and HO_2_ compared to the other structures, indicating its stronger capability to capture H_2_O_2_ and related free radicals to prevent them from damaging the active sites (Figures 1c; S2, Supporting Information). Furthermore, when the concentration of *V*_O_ reaches a certain threshold (10%), the adsorption strength is maximized, and any further increase in oxygen vacancies does not notably enhance the adsorption capacity (Figures 1d–h; S3, Supporting Information). Generally, these calculation results clearly demonstrate that valence engineering in optimizing the oxidation state of CeO_2_ can significantly enhance its efficiency in scavenging the generated H_2_O_2_ and other free radicals, thereby substantially reducing the damage caused to the FeCo─N─C.

### 2.2 Morphology and Structure Characterizations

**Figure 2:**
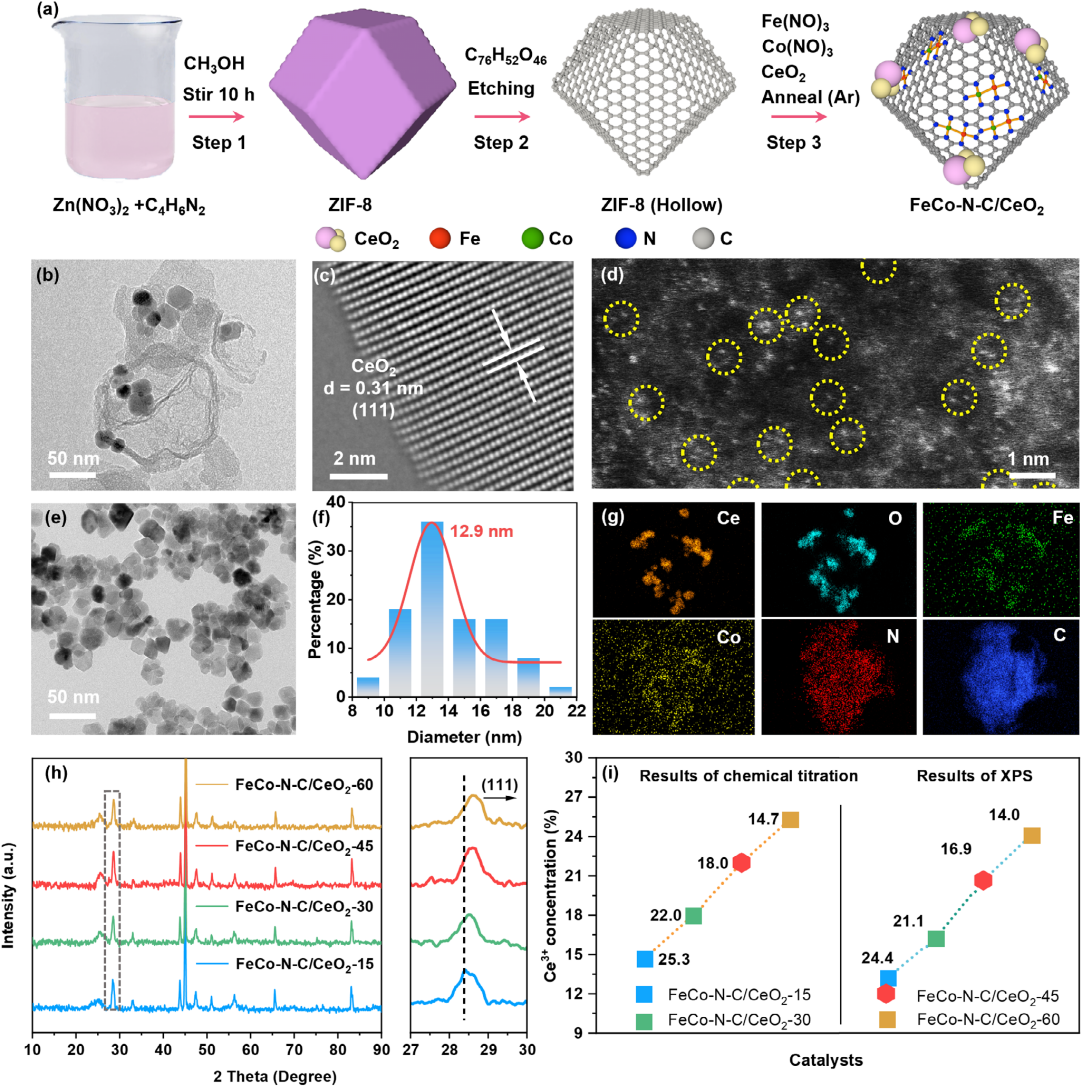
Structural and morphological characterizations of FeCo─N─C/CeO_2_ catalysts. a) Schematic illustration for the fabrication procedure of FeCo─N─C/CeO_2_. b) TEM, c) STEM, and d) HAADF‐STEM images of FeCo─N─C/CeO_2_‐45. e) TEM image of CeO_2_‐45. f) The particle‐size distribution of CeO_2_‐45 obtained from Figure 2e. g) TEM‐EDS elemental mappings of Ce, O, Fe, Co, N, and C in FeCo‐N‐C/CeO_2_‐45. h) XRD patterns of FeCo─N─C/CeO_2_‐X (X = 15, 30, 45, and 60). i) The variation in Ce^3+^ concentration in FeCo─N─C/CeO_2_‐X with increasing NaBH_4_ etching time was determined by chemical titration (left) and XPS analysis (right), respectively.

**Figure** **2**a depicts the preparation process of the FeCo─N─C/CeO_2_ catalyst. The hollow‐structure ZIF‐8 was first synthesized by a templating method, which was subsequently combined with iron nitrate, cobalt nitrate, and CeO_2_‐X (X = 15, 30, 45, and 60, referring to the soaking time in NaBH_4_ solution in minutes) by immersion. Ultimately, high‐temperature annealing was conducted to fabricate the FeCo─N─C/CeO_2_‐X composites. Scanning electron microscopy (SEM) and transmission electron microscopy (TEM) images show that FeCo─N─C displays a similar hollow cage‐like structure as ZIF‐8 (Figure S4a–c, Supporting Information), with a well‐defined morphology and a size of ≈100 nm. FeCo─N─C/CeO_2_ composite (Figures 2b; S4d, Supporting Information) reveal that the CeO_2_ particles are nicely mixed with hollow FeCo─N─C. The formation of the hollow structure is attributed to the precise design of the MOF template and controlled temperature during the annealing process.^[^ ^30^ ^]^ This morphology can effectively expose more active sites and provide a platform for loading additional CeO_2_ nanoparticles.

The microscopic structure and composition of FeCo─N─C/CeO_2_‐X (X = 15, 30, 45, 60) were further studied by TEM. Low‐magnification TEM images revealed that CeO_2_‐X particles of different treatments were dispersed with the hollow cage‐like FeCo─N─C, without significant difference in the overall morphology (Figure S5, Supporting Information). The edges of hollow structures remain clearly visible, with no morphological collapse or damage, emphasizing the structural stability and resilience of the ZIF‐8‐derived cage‐like structure. High‐resolution TEM (HRTEM) images of FeCo─N─C/CeO_2_‐45 at the well crystalline region (Figure 2c) showed two interplanar spacings of 0.19 and 0.13 nm, corresponding to the (220) and (400) planes of CeO_2_, respectively. Atomic‐scale dispersion of dual‐atom sites and a small number of metal clusters were observed in FeCo─N─C/CeO_2_‐45 using spherical aberration‐corrected scanning transmission electron microscopy high‐angle annular dark‐field (STEM‐HAADF) images (Figures 2d; S6a,b, Supporting Information). The selected area electron diffraction (SAED) pattern (Figure S6c, Supporting Information) displayed two sets of diffraction rings, attributed to the (111), (113), (004), (044), and (531) planes of CeO_2_, and the (002) plane of carbon, respectively.^[^ ^31^ ^]^ In addition, CeO_2_ nanoparticles have an average size of 12.9 nm (Figures 2e,f; S7, Supporting Information). TEM‐EDS elemental mapping (Figures 2g; S8, Supporting Information) confirmed that the Ce and O elements were localized on the CeO_2_ particle attached to the hollow structure, while the Fe and Co elements were distributed across the nitrogen‐doped carbon framework. These analyses suggest that CeO_2_ nanoparticles were successfully embedded into the FeCo─N─C hollow structure.

The structural properties of the samples obtained at each stage were confirmed through X‐ray diffraction (XRD) patterns (Figures 2 h; S9, Supporting Information). The crystal structure of the ZIF‐8 precursor remained unchanged after tannic acid etching (Figure S9a, Supporting Information). After the introduction of a minimal quantity of metal atoms and subsequent annealing, diffraction peaks associated with carbon and Co_0.72_Fe_0.28_ (JCPDS no. 51–0740) were detected in FeCo─N─C sample (Figure S9c, Supporting Information). The XRD pattern of CeO_2_ exhibited good crystallinity (Figure S9b, Supporting Information). The FeCo─N─C/CeO_2_ composite exhibited diffraction peaks indicative of both FeCo─N─C and CeO_2_. For the composite materials with FeCo─N─C and CeO_2_‐X (Figure 2h), the CeO_2_ diffraction peaks gradually shifted to higher angles with increased soaking time in sodium borohydride (NaBH_4_), suggesting a decrease in the lattice spacing. This change could be attributed to the emergence of *V*_O_ in CeO_2_ caused by the NaBH_4_ treatment.^[^ ^32^ ^]^

The proportion of Ce^3+^ in the samples was determined by quantifying the percentage of Ce^3+^ relative to the total Ce content using a chemical titration (Figures 2i; S10, Supporting Information). Specifically, CeO_2_ was dissolved in a 6 mol L^−1^ HNO_3_ solution, and 30 mL of 30% H_2_O_2_ was added to reduce all the Ce^4+^ to Ce^3+^,^[^ ^33^ ^]^ which served as the 100% standard reference. In the solution, Ce^3+^ appears yellow and reacts with KMnO_4_ to decolorize its purple color, but Ce^4+^ remains white and does not react with KMnO_4_. The measured volume of 0.02 mol L^−1^ KMnO_4_ consumed during titration was used to compute the Ce^3+^ level in the samples. The volume of KMnO_4_ required to achieve decolorization for FeCo─N─C/CeO_2_ was 150 mL. In comparison, for FeCo─N─C/CeO_2_‐15, FeCo─N─C/CeO_2_‐30, FeCo─N─C/CeO_2_‐45, and FeCo─N─C/CeO_2_‐60, the volumes of KMnO_4_ consumed during titration were 22, 27, 33, and 38 mL, respectively. According to the stoichiometric relationship of the reaction, the proportions of Ce^3+^ in reference to the total Ce content in the samples were determined to be 14.7% (FeCo─N─C/CeO_2_‐15), 18.0% (FeCo─N─C/CeO_2_‐30), 22.0% (FeCo─N─C/CeO_2_‐45), and 25.3% (FeCo─N─C/CeO_2_‐60), respectively.

The efficiency of catalytic reactions is significantly affected by the active sites on the catalyst surface layer.^[^ ^34^ ^]^ Therefore, the surface elemental composition was investigated using X‐ray photoelectron spectroscopy (XPS). The XPS full spectra confirmed the presence of the expected elements, including C, N, Co, and Fe for the FeCo─N─C, and O and Ce for the CeO_2_‐X (Figure S11, Supporting Information). Analysis of the C 1*s* spectrum revealed the presence of C─N bonds (Figure S12a,b, Supporting Information), confirming the successful formation of nitrogen‐doped carbon in the hollow structure.^[^ ^35^, ^36^ ^]^ The N 1*s* spectra (Figure S12c,d, Supporting Information) revealed that the proportions of different nitrogen species, namely pyridine nitrogen, pyrrole nitrogen, graphitic nitrogen, and oxidized nitrogen,^[^ ^37^, ^38^ ^]^ are similar in different FeCo─N─C/CeO_2_‐X catalysts (Table S1, Supporting Information). The O 1*s* spectra (Figure S13a, Supporting Information) showed that the oxygen species mainly consisted of metal‐oxygen bonds, O vacancies, and adsorbed oxygen.^[^ ^39^ ^]^ As prolonging the soaking time for CeO_2_, the metal‐oxygen bond signal decreased, while the proportion of O vacancies increased from 56.1% in FeCo‐N‐C/CeO_2_‐15 to 71.8% in FeCo‐N‐C/CeO_2_‐60 (Figure S13c, Supporting Information). Since the XPS is surface sensitive, the high deconvoluted percentage only suggested that the O vacancies are concentrated on the surface of CeO_2_. While the bulk of CeO_2_ remains in good crystallinity, as evidenced by the XRD characterization (Figure S9d, Supporting Information). In the Ce 3*d* spectra (Figure S13b, Supporting Information), peaks A, C, D, as well as A', C', and D' correspond to the electronic orbitals of Ce^4+^ in CeO_2_, while peaks B and B' are attributed to Ce^3+^ orbitals.^[^ ^26^ ^]^ As increasing the soaking time in NaBH_4_, the proportion of Ce^3+^ among the total cerium species improved from 14.02% in FeCo─N─C/CeO_2_‐15 to 24.39% in FeCo─N─C/CeO_2_‐60 (Figure S13c, Supporting Information), indicating the formation of O vacancies in CeO_2_ by reducing Ce^4+^ to Ce^3+^. The increasing trend of Ce^3+^ by prolonging the soaking time in NaBH_4_ verified by XPS characterizations, is in accordance with the result obtained from chemical titration.

In addition, the Fe and Co K‐edge X‐ray absorption near‐edge structure (XANES) spectra have been collected to understand their chemical environments (Figure S14a,b, Supporting Information). The Co‐K and Fe‐K absorption edge positions of the FeCo─N─C sample are clearly shifted to higher energies compared with those of Fe foil and Co foil, indicating slightly higher average oxidation states. In the Fourier transform extended X‐ray absorption fine structure (FT‐EXAFS) spectra (Figure S14c, Supporting Information), weak signals for Fe─N (≈1.42 Å) and Co─N (≈1.37 Å) bonds are present. Since the diffraction peaks of the Co_0.72_Fe_0.28_ phase are observed in the XRD pattern and atomic pairs are observed in the HAADF‐STEM images, it is clear that FeCo clusters and atomically dispersed Fe‐Co species are both present in the sample.

Furthermore, the effects of CeO_2_ on the FeCo─N─C structure were further studied using Raman spectroscopy (Figure S15, Supporting Information). The 466 cm^−1^ peak results from CeO_2_ vibrations, while the peaks at 1350 and 1590 cm^−1^ matched the D‐band (structural defects) and G‐band (graphitic structure)^[^ ^40^ ^]^ of FeCo─N─C, respectively. The values of I*_D_*/I*_G_* remain similar when incorporated with different CeO_2_‐X nanoparticles, showing that structural defects and catalytic graphitization of the carbon‐matric were unaffected. Moreover, the nitrogen adsorption‐desorption results (Figure S16, Supporting Information) showed that the introduction of CeO_2_ reduced the specific surface area of the composite compared to that of the FeCo─N─C; while the pore size remained within the mesoporous range, further proving the hollow structure is not destroyed by introducing CeO_2_.

### 2.3 Oxygen Electrocatalysis Performance

**Figure 3:**
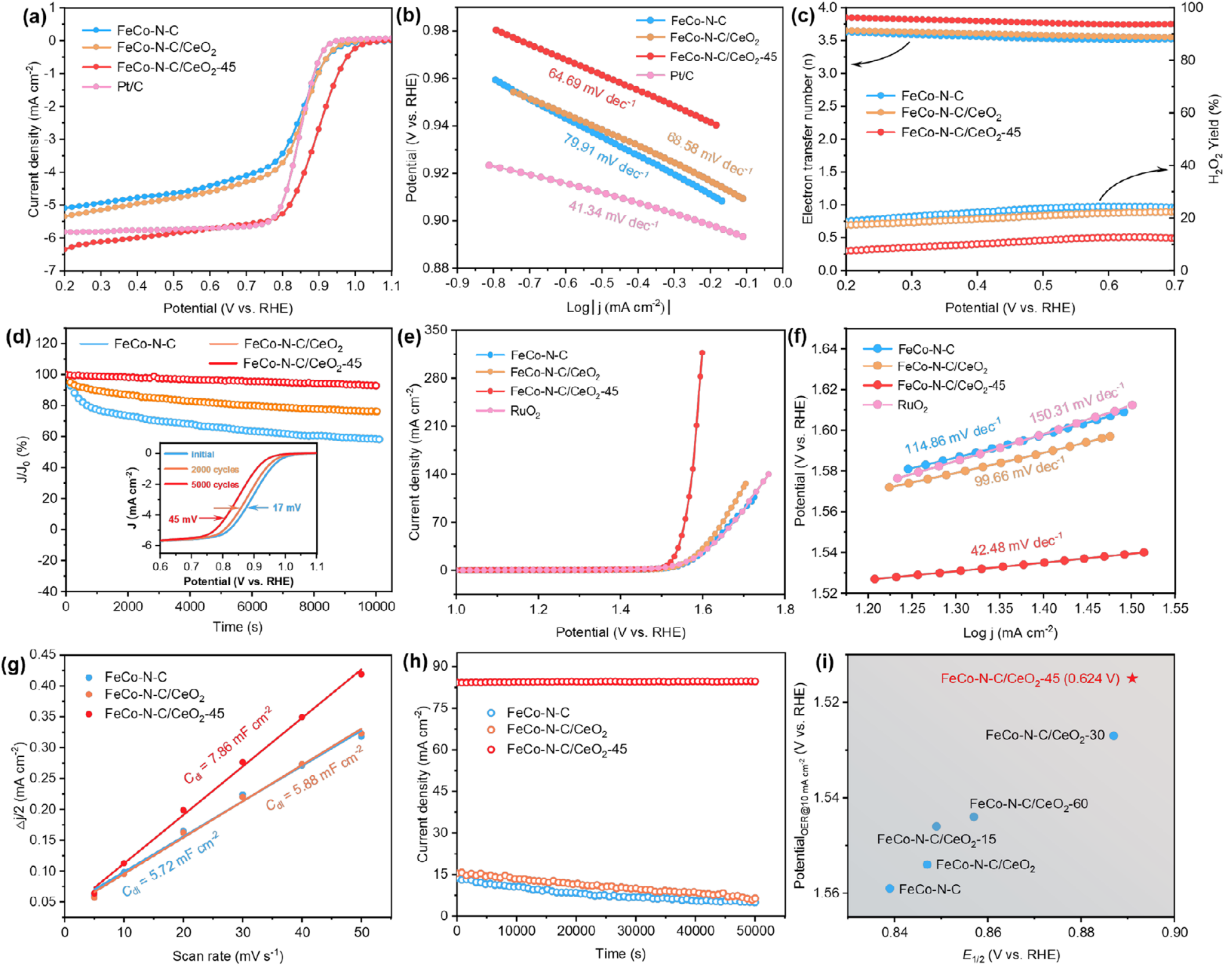
Oxygen electrocatalytic performance of FeCo─N─C, FeCo─N─C/CeO_2_ and FeCo─N─C/CeO_2_‐45. a) LSV curves of ORR recorded in O_2_‐saturated KOH electrolyte, b) the corresponding Tafel plots, and c) the electron transfer number and H_2_O_2_ yield calculated from the LSV curves (Figure S14a,b, Supporting Information). d) The *i*–*t* curves in O_2_‐saturated 0.1 m KOH electrolyte, and the insets show LSV curves recorded before and after 2000 and 5000 cycles of FeCo─N─C/CeO_2_‐45. OER performance evaluated in terms of e) the LSV curves recorded in O_2_‐saturated electrolyte and f) the corresponding Tafel plots. g) The current density difference at 1.075 V vs RHE is plotted as a function of scan rate to extract the *C*_dl_. h) The *i*–*t* curves recorded at 1.58 V vs RHE. i) Comparison of the potential difference between *E*_1/2_ for ORR and *E*_j = 10_ for OER.

The influences of combining CeO_2_ with FeCo─N─C on catalytic activity were initially assessed by evaluating the ORR performance of the synthesized catalysts. The introduction of CeO_2_ with FeCo─N─C resulted in an anodic shift of the reduction peak potential than FeCo─N─C (Figure S17a, Supporting Information).^[^ ^41^ ^]^ Furthermore, as the Ce^3+^ content increased, the potential initially increased, subsequently declined, peaking at a Ce^3+^ content of ≈22.0% in FeCo─N─C/CeO_2_‐45 (Figure S17b, Supporting Information). The LSV curves show that the trend of ORR activity after combining different CeO_2_‐X with FeCo─N─C is consistent with the CV results (**Figures** **3**a; S18 and S19a, b, Supporting Information). Among the as‐prepared catalysts, FeCo─N─C/CeO_2_‐45 exhibited the highest onset potential (*E*_onset_ = 1.024 V) and half‐wave potential (*E*_1/2_ = 0.890 V), even surpassing those of Pt/C (*E*_onset_ = 1.000 V and *E*_1/2_ = 0.849 V).^[^ ^42^, ^43^ ^]^ Additionally, its Tafel slope was 64.69 mV dec^−1^ (Figures 3b; S19c, Supporting Information). Overall, compared to other prepared catalysts, FeCo─N─C/CeO_2_‐45 shows the highest electron transfer number (average *n* = 3.81) and the lowest H_2_O_2_ yield (≈9.95%), indicating that the 4‐electron ORR pathway is more favorable and thus the H_2_O_2_ yield was significantly reduced during the reaction (Figures 3c; S19 d–f, Supporting Information).^[^ ^44^ ^]^ In chronoamperometry tests performed at 0.7 V vs. RHE, the current density remained at 92% of its initial value after 10 000 s (Figures 3d; S20, Supporting Information). Due to its inherent scavenging properties, CeO_2_ facilitates the conversion of H_2_O_2_ and other oxidative radicals. Therefore, combining the oxidation valence‐engineered CeO_2_ with FeCo─N─C can enhance the durability of the catalyst.

The activity of FeCo─N─C in the OER is comparatively constrained due to the difficulty in rapidly altering the oxidation state of its active sites during the reaction process.^[^ ^45^ ^]^ We show that the incorporation of CeO_2_ and CeO_2_‐*V*_O_ with poor OER activity itself can improve OER performance of FeCo─N─C (Figure S21, Supporting Information). The enhancement becomes more evident by valence engineering in CeO_2_. Specifically, FeCo─N─C/CeO_2_‐45 exhibited excellent OER catalytic activity (Figure 3e), with the smallest overpotential (*η*_10_) of only 285 mV. More importantly, in Figures 3f and S21c (Supporting Information), the Tafel slope of FeCo─N─C was 114.8 mV dec^−1^ indicating relatively slow OER kinetics;^[^ ^46^ ^]^ in contrast, the Tafel slope (FeCo─N─C/CeO_2_‐45) was significantly reduced to 42.48 mV dec^−1^, with accelerated OER kinetics. In addition, the double‐layer capacitance (*C*_dl_) of FeCo─N─C/CeO_2_‐45 is 7.86 mF cm^−2^, also larger than the 5.72 mF cm^−2^ for FeCo─N─C (Figures 3 g; S22–S24, Supporting Information). Chronoamperometric tests performed at 1.58 V demonstrated that FeCo─N─C/CeO_2_‐45 exhibited excellent stability during long‐term continuous operation (Figures 3 h; S25, Supporting Information). For bifunctional oxygen electrocatalysts, the potential difference (△*E*) between the *E*_1/2_ of the ORR and the *η*_10_ of the OER is a crucial indicator of performance.^[^ ^47^ ^]^ As shown in Figure 3i, FeCo─N─C/CeO_2_‐45 exhibited a △*E* of 0.625 V, making it one of the most efficient oxygen electrocatalysts and demonstrating its great potential for application in ZABs (Table S2, Supporting Information).

### 2.4 Mechanistic Insights into the Stability and Activity Enhancement

**Figure 4:**
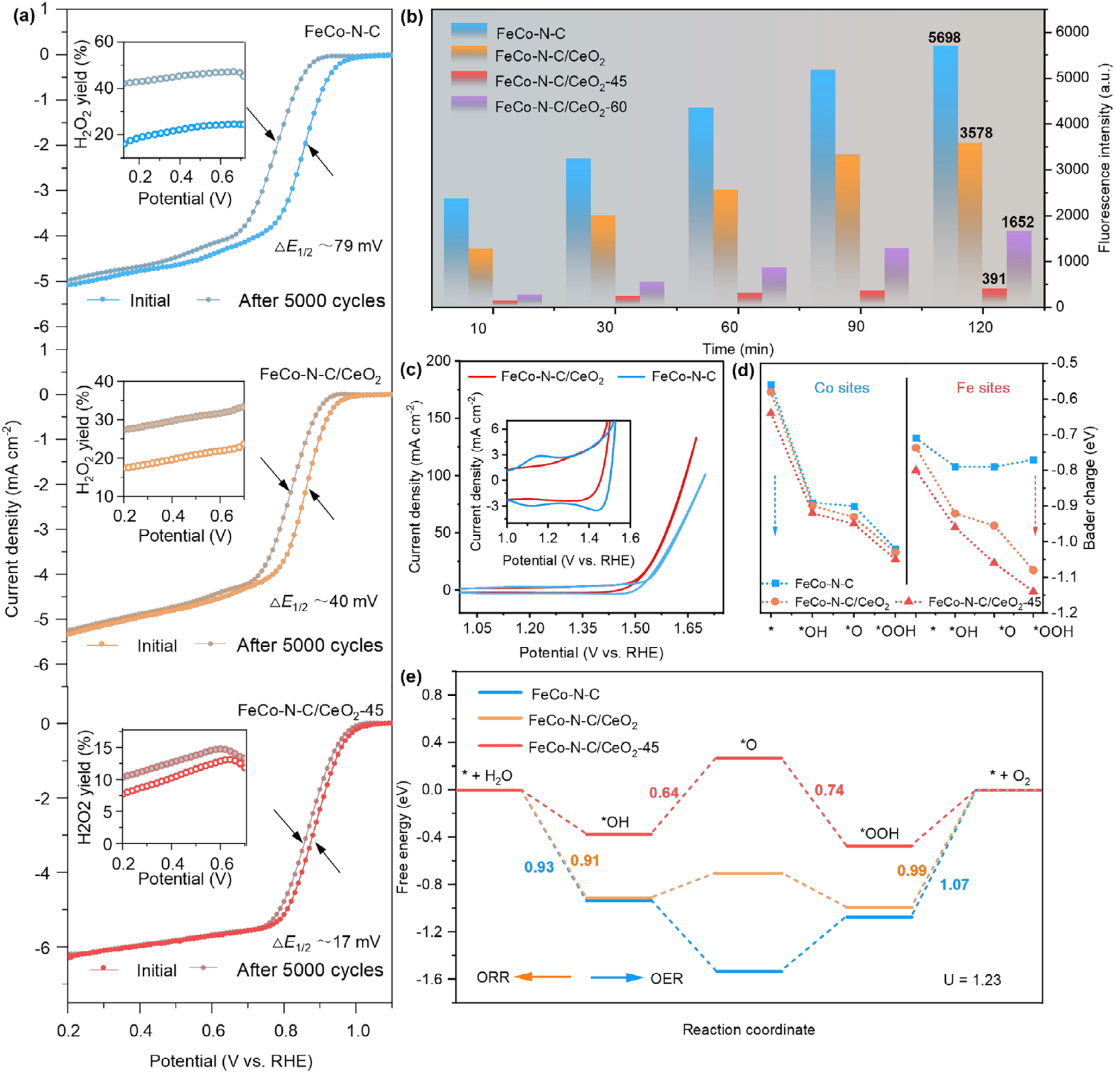
Mechanistic insights into the enhanced ORR/OER performance. a) The ORR performance of FeCo─N─C, FeCo─N─C/CeO_2_, and FeCo─N─C/CeO_2_‐45 catalysts before and after 5000 CV cycles. The insets show the corresponding H_2_O_2_ yield as a function of applied potential. b) The measured fluorescence intensity as an indicator for Radical elimination in different catalysts. c) Comparison of CV curves of FeCo─N─C and FeCo─N─C/CeO_2_ in 1.0 m KOH, with an inset showing a magnified view. d) The Bader charge values of Co (left) and Fe (right) sites at each step of the OER process, and e) free energies of the intermediates for the OER (left to right) and ORR (right to left) in the FeCo─N─C, FeCo─N─C/CeO_2_, and FeCo─N─C/CeO_2_‐45 catalysts.

To systematically evaluate the impact of CeO_2_ introduction and valence engineering on the catalyst's durability, we performed CV tests for 5000 cycles on FeCo─N─C, FeCo─N─C/CeO_2_, and FeCo─N─C/CeO_2_‐45 catalysts at 0.6–1.0 V and 20 mV s^−1^. We note that potentials above 1.0 V can cause carbon corrosion and irreversible structural damage. Therefore, the 0.6–1.0 V range was adopted as the upper limit to balance the electrochemical rigor, structural integrity, and data reliability. The activity of FeCo─N─C significantly decreased after cycling, with its *E*_1/2_ shifting negatively by 79 mV compared to the initial state (**Figure** **4**a). Moreover, at 0.7 V, its H_2_O_2_ yield increased to 48%, approximately twice the initial value. This unusually high H_2_O_2_ yield can be primarily attributed to the degradation of metal–N*_x_* sites during the reaction. In contrast, FeCo─N─C/CeO_2_ and FeCo─N─C/CeO_2_‐45 exhibited significantly better performance in durability. Specifically, the *E*_1/2_ value for FeCo─N─C/CeO_2_‐45 was negatively shifted by only 17 mV, while ≈40 mV shift was observed in that of FeCo─N─C/CeO_2_. At 0.7 V vs RHE, the H_2_O_2_ yield of FeCo─N─C/CeO_2_‐45 remained at ≈13%, while that of FeCo─N─C/CeO_2_ is increased from 22% to 33%, both lower than the H_2_O_2_ yield of the FeCo─N─C.

To quantitatively analyze the effect of CeO_2_ on free radical scavenging efficiency, we monitored the changes in free radical concentration using fluorescence spectroscopy (Figures 4b; S26, Supporting Information). Coumarin was selected as the fluorescent probe, reacting with free radicals to form 7‐hydroxycoumarin, which exhibits strong fluorescent properties.^[^ ^48^ ^]^ Using Fenton's reagent as the free radical source, we compared the fluorescence intensity of the Fenton reagent/FeCo─N─C, Fenton reagent/FeCo─N─C/CeO_2_, Fenton reagent/FeCo─N─C/CeO_2_‐45, and Fenton reagent/FeCo─N─C/CeO_2_‐60 systems over a reaction time of 10–120 min. The intensity of the fluorescence follows the trend of FeCo─N─C > FeCo─N─C/CeO_2_ > FeCo─N─C/CeO_2_‐60 >FeCo─N─C/CeO_2_‐45, indicating the concentration of free radicals is the lowest in the system with FeCo─N─C/CeO_2_‐45, highlighting its excellent capability in free radical scavenging.

In the FeCo─N─C system, the fluorescence intensity remained at 5698 after 120 min. With the introduction of CeO_2_, the fluorescence intensity of the FeCo─N─C/CeO_2_ system decreased to 3578 after 120 min, primarily due to the reaction between Ce^4+^ in CeO_2_ and OOH as well as H_2_O_2_, which partially degraded free radicals. However, a certain amount of ·OH remained in the system. In contrast, in the FeCo─N─C/CeO_2_‐45 system, CeO_2_‐45 contained not only Ce^4+^ but also a certain proportion of Ce^3+^. The presence of Ce^3+^ further facilitated the conversion of ·OH, significantly reducing the hydroxyl radical concentration, leading to a fluorescence intensity of 391 after 120 min. However, as the Ce^3+^ content further increased (i.e., in the FeCo‐N‐C/CeO_2_‐60 system), the relative content of Ce^4+^ decreased, impairing its ability to consume ·OOH and H_2_O_2_, thereby increasing the fluorescence intensity to 1652. CeO_2_ played a crucial regulatory role in free radical degradation; however, the mere introduction of CeO_2_ was insufficient to achieve optimal free radical scavenging efficiency. The modulation of the Ce^4+^/Ce^3+^ redox state significantly influenced the catalytic system's ability to eliminate different types of free radicals. Therefore, precise control of the Ce^4+^/Ce^3+^ ratio is essential for optimizing the free radical scavenging capacity of the catalyst, indicating that the valence‐engineering strategy offers greater optimization potential than the mere introduction of CeO_2_.

In addition, the significant difference in OER catalytic performance between FeCo─N─C and FeCo─N─C/CeO_2_ is clearly demonstrated by CV experiments conducted in a 1 M KOH (Figure 4c). FeCo─N─C catalyst exhibits obvious redox peaks, attributed to the redox processes of Co active sites during the OER process. In contrast, in FeCo─N─C/CeO_2_, the redox peaks and the OER onset is lower compared to those in FeCo─N─C. This suggests that the addition of CeO_2_ significantly modulates the redox properties of Co in the FeCo─N─C. To further investigate the pre‐OER redox mechanism of FeCo─N─C and its correlation with catalytic activity, we performed a pH‐dependent analysis of its reduction peak within a solution pH range of 12.8 to 14.0. The potential of the reduction peak relative to the SHE was plotted against solution pH (Figure S27a, Supporting Information), to extract a slope of 90.0 ± 3.3 mV per pH indicative of a 2 e^−^/3 H^+^ redox process. Furthermore, CV curves of FeCo─N─C in 1 m KOH at different scan rates show a quasi‐first‐order power law for decreasing peak current density (Figure S27b, Supporting Information). This suggests that the reduction peak is closely associated with the surface capacitance process that not limited by the mass transport.^[^ ^49^, ^50^ ^]^ In situ electrochemical impedance spectroscopy (EIS) results (Figure S28, Supporting Information) indicate that the reduction of charge‐transfer resistance (R_ct_) in FeCo─N─C/CeO_2_ catalysts is substantially more pronounced compared to the FeCo─N─C, suggesting that the introduction of CeO_2_ markedly enhances interfacial charge‐transfer efficiency.

For the OER process, both Co_0.72_Fe_0.28_ and FeCo─N─C structures can potentially serve as active sites. Therefore, the Gibbs free energies required for the OER on these two structures were calculated. Since the strongest XRD peak of Co_0.72_Fe_0.28_ corresponds to the (420) crystal plane, the slab was constructed along the (420) plane. During the OER process (from left to right) (Figures S29 and S30, Supporting Information), the highest energy barrier on Co_0.72_Fe_0.28_ reaches 1.91 eV, substantially exceeding the 1.07 eV observed on FeCo─N─C. In comparison, CeO_2_ and CeO_2_‐*V*_O_ exhibit even larger rate‐determining barriers of 2.11 and 2.07 eV, respectively. These results indicate that the OER is energetically more favorable at the FeCo─N─C sites. Conversely, considering the ORR process (from right to left), the energy required for ORR on Co_0.72_Fe_0.28_ is also higher. Generally, the FeCo‐N‐C shows a better lower thermodynamic barrier than Co_0.72_Fe_0.28_, CeO_2_, and CeO_2_‐*V*_O_ for both ORR and OER. Through the analysis of the Bader charge distribution changes at Fe and Co sites in FeCo─N─C, FeCo─N─C/CeO_2_, and FeCo─N─C/CeO_2_‐45 during OER process (Figures 4d; S30, Supporting Information), we systematically evaluated the charge transfer characteristics and oxidation state changes in the systems. In the Bader charge analysis, negative values indicate electron loss. The Fe and Co sites in the FeCo─N─C/CeO_2_ system exhibited greater electron loss during the ^*^OH, ^*^O, and ^*^OOH intermediate stages, indicating more pronounced oxidation state changes than FeCo─N─C. FeCo─N─C/CeO_2_‐45 demonstrated the highest degree of electron loss, corresponding to the most significant oxidation transitions of Fe and Co, thereby exhibiting enhanced OER activity. The electrons lost from Fe and Co sites combined with Ce^4+^ in CeO_2_, with CeO_2_ acting as an electron acceptor.

To understand the mechanism by which variations in the Ce valence ratio enhance the oxidation transition capability of Fe and Co during the OER, we measured the bandgap of CeO_2_‐X (X = 0, 15, 30, 45, and 60) (Figure S31, Supporting Information). As the NaBH_4_ immersion time increased, the bandgap of the samples consistently decreased, indicating improved conductivity with the rise in Ce^3+^ content. When the Ce^3+^ content reached ≈20%, the synergic effects of improved conductivity and Ce^3+^/Ce^4+^ conversion are optimized, resulting in the best OER performance. However, further increases in Ce^3+^ content reduced the availability of Ce^4+^ in CeO_2_ for electron binding, leading to a decline in electron absorption capability. Thus, a balance between conductivity and Ce^4+^ availability is key for FeCo─N─C/CeO_2_‐45 to deliver optimal OER activity.

To compare the activity differences of the three catalysts in both the OER and ORR, we calculated their reaction free energy (Figure 4e). In OER, the rate‐determining step (RDS, which is determined by the step with the highest energy barrier) for FeCo─N─C is the conversion of OOH to O_2_, with a corresponding free energy change (△*G*) of 1.07 eV. The introduction of CeO_2_ facilitates the deprotonation process, lowering the free energy required for this conversion to 0.99 eV. By optimizing the valence state ratio in CeO_2_, the energy required for this step is further reduced to 0.37 eV, and the RDS shifts from the O to OOH conversion to the deprotonation of OH with a lower energy of 0.64 eV. Therefore, the overall free energy profile shows that FeCo─N─C/CeO_2_‐45 has the lowest energy barrier in the RDS, resulting in the best OER activity. This phenomenon aligns with the previous Bader charge analysis, suggesting that CeO_2_ enhances OER activity by promoting faster valence state changes, resulting in lower energy requirements for FeCo─N─C/CeO_2_‐45 in the OER. In ORR pathway, FeCo─N─C/CeO_2_‐45 also shows lower free energy than the other two catalysts, with the RDS shifting from the ^*^OH deprotonation process in FeCo─N─C (0.93 eV) and FeCo─N─C/CeO_2_ (0.91 eV) to the ^*^OOH dehydroxylation process (0.74 eV). Thus, valence engineering in the CeO_2_ redox modulator simultaneously enhances both OER and ORR performance.

### 2.5 ZABs Performance

**Figure 5:**
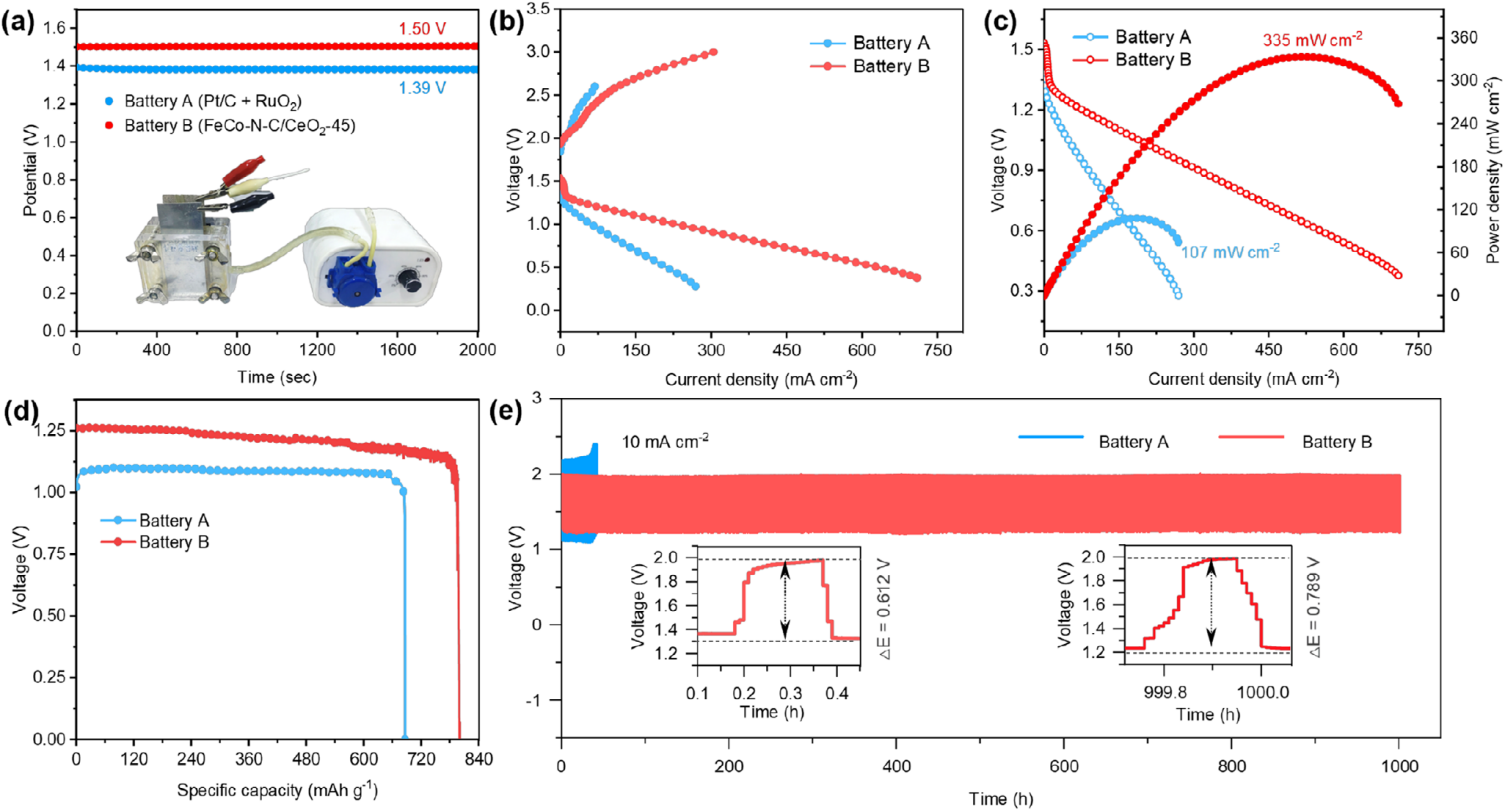
FeCo─N─C/CeO_2_‐45 performance in rechargeable ZABs. a) Open‐circuit potential of Pt/C+ RuO_2_ (Battery A) and FeCo─N─C/CeO_2_‐45 (Battery B). The inset shows a photograph of the fabricated ZABs. b) Charging‐discharging polarization plots of the batteries in the air electrodes. c) Discharge curves and the corresponding power density curves. d) Galvanostatic discharge curves at 10 mA cm^−2^. The specific capacity was calculated based on the mass lost due to Zn consumption. e) Galvanostatic charging/discharging cycling curves at 10 mA cm^−2^; the insets show a minimal change in the potential gap after cycling for 1,000 h.

The FeCo─N─C/CeO_2_‐45 bifunctional catalyst was loaded onto a nickel foam to fabricate the air electrode for a ZAB (**Figure** **5**a, Battery B). For comparison, a battery using mixed catalysts (Pt/C and RuO_2_) was also tested (Battery A, control sample). Battery B exhibited an open‐circuit voltage of 1.50 V, significantly higher than the 1.39 V recorded for Battery A. Besides, the voltage polarization curve of Battery B showed a smaller gap, further demonstrating its superior charging performance (Figure 5b). The maximum power density of Battery B reached 335 mW cm^−2^, far surpassing the 107 mW cm^−2^ of Battery A, and outperformed most ZABs systems reported in the literature (Figure 5c). In terms of specific capacity, Battery B exhibited a value of 801 mAh g^−1^ at 10 mA cm^−2^, ≈15% higher than that of Battery A (Figure 5d). Furthermore, in the continuous charge‐discharge tests lasting over 1000 h (Figure 5e), Battery B maintained a round‐trip efficiency of over 60%. At higher current densities of 20 and 50 mA cm^−2^, the cell maintained stable round‐trip efficiency for 680 and 540 h (Figure S32, Supporting Information), respectively, demonstrating its excellent stability and reversibility in oxygen electrocatalysis. After 1000 h of continuous charge/discharge cycling, the zinc anode surface remained generally smooth, exhibiting only slight local roughening without any obvious long needle‐like dendrites or large‐area irreversible deposits (Figure S33, Supporting Information). This indicates that the gradual performance decay primarily originates from the progressive deactivation of the catalytically active sites on the air electrode, rather than morphological changes of the zinc anode.

Furthermore, XPS analysis (Figure S34, Supporting Information) after cycling was used to study the changes in Ce oxidation state after long‐term operation in the FeCo‐N‐C/CeO_2_‐X system. During the ORR process, Ce^4+^ is reduced to Ce^3+^, by effectively scavenging the H_2_O_2_ and ·OOH, while Ce^3+^ can combine with ·OH to convert back to Ce^4+^. In the early stages of ORR, the Ce^3+^ content is 21.1%, and the scavenging effect was most significant. After 5000 CV cycles, the proportion of Ce^3+^ in the FeCo‐N‐C/CeO_2_‐45 catalyst was 20.9%, showing a negligible change compared to before cycling, indicating that the Ce^4+^/Ce^3+^ interconversion is under a near‐dynamic equilibrium. For the OER process, a 50 000 s constant potential test at 1.58 V was performed on the FeCo‐N‐C/CeO_2_‐45 catalyst. After testing, the proportion of Ce^3+^ was 22.5%, also close to that before the test, supporting that CeO_2_‐45 can act as an electronic modulator to undergo reversible oxidation state transitions. Further assembling the catalyst into a ZABs for 100 h of charge‐discharge testing (OER followed by ORR), the proportion of Ce^3+^ in the battery remained almost identical to that before testing, further verifying the stability and reliability of this regulator in practical applications. Overall, after ORR and OER reactions, the proportion of Ce^3+^ in FeCo─N─C/CeO_2_‐45 remained ≈21.1%, indicating that valence‐engineered CeO_2_ is a durable redox modulator.

## 3 Conclusion

In summary, air electrodes in ZABs face two principal challenges in practical applications: inadequate durability resulting from the attack of highly oxidative by‐products on the active sites during ORR, and the limited OER activity constrained by poor oxidation‐state variability in their inherent structure. To address these issues, we introduced the CeO_2_ redox modulator with the FeCo─N─C dual‐atom catalyst. The valence engineering in CeO_2_ can maximize its capability in tuning the oxygen electrocatalysis. On the one hand, optimizing the Ce^3+^/Ce^4+^ ratio enables the fast redox transformation to scavenge H_2_O_2_ and other free radicals to reduce the active site degradation in ORR process. On the other hand, the CeO_2_ modifies the electronic density around the Fe and Co sites, thereby tuning their redox properties and improving the OER activity. As a result, the assembled ZABs with the optimized catalyst achieved a peak power density exceeding 335 mW cm^−2^ and demonstrated excellent stability after continuous cycles for more than 1000 h. This study successfully proves the valence engineering of CeO_2_ redox modulator as a practical method to overcome the limitation in dual‐atom catalysts, offering new insight for designing efficient oxygen electrocatalysts for ZABs and other related applications.

## 4 Experimental Section

### Preparation of Hollow ZIF‐8

Zn(NO_3_)_2_·6H_2_O, 4.464 g, was dissolved in 120 mL of methanol and stirred until it was homogeneous. Subsequently, 4.928 g of 2‐methylimidazole was dissolved in 120 mL methanol and added to the above solution, followed by stirring for 6 h. The obtained product was separated by centrifugation to obtain ZIF‐8. Next, 100 mg of ZIF‐8 was distributed in 50 mL of methanol containing 250 mg of tannic acid. After stirring for 5 min, the product was separated by centrifugation to obtain the hollow ZIF‐8 sample.

### Preparation of FeCo─N─C/CeO_2_‐X (X = 15, 30, 45, and 60)

Eighty mg of hollow ZIF‐8 was dispersed in 13 mL of n‐hexane and sonicated for 1 h. Subsequently, 50 mL of 20 mg mL^−1^ Fe(NO_3_)_3_·9H_2_O and Co(NO_3_)_2_·6H_2_O solutions, along with 50 mL of 10 mg mL^−1^ CeO_2_‐X suspension (The preparation process is detailed in the Supporting Information), were added dropwise to the solution. The mixture was then sonicated to form a homogeneous suspension. After stirring the suspension for 2 h, it was collected by centrifugation. Finally, the dried powder was placed in a tube furnace, heated to 900 °C at 5 °C min^−1^ under a 5% Ar‐H_2_ mixed gas atmosphere, and annealed for 1 h, yielding the FeCo‐N‐C/CeO_2_‐X sample.

### Computational Methods

The DFT was used as implemented in the Vienna Ab initio simulation package (VASP) in all calculations. The exchange‐correlation potential was described by using the generalized gradient approximation of Perdew‐Burke‐Ernzerhof (GGA‐PBE). The projector augmented‐wave (PAW) method was employed to treat interactions between ion cores and valence electrons. The plane‐wave cutoff energy was fixed to 450 eV. Given structural models were relaxed until the Hellmann‐Feynman forces smaller than −0.02 eV Å^−1^ and the change in energy smaller than 10^−5^ eV was attained. Grimme's DFT‐D3 methodology was used to describe the dispersion interactions among all the atoms in adsorption models. The Gamma‐centered k‐points samplings were set to 2 × 1 × 1 for the model. The vacuum space along the *z*‐direction was set to be 12 Å.

Based on the XRD and TEM results, the (111) crystal plane was selected as the cleavage surface for CeO_2_ model construction. The (111) surface was generated using the Cleave Surface function in Material Studio, with a thickness of four atomic layers and a vacuum layer of 15 Å. The atomic ratio of Ce to O was set to 1: 2. Using the scripting function in Material Studio, CeO_2_ models containing 5%, 10%, and 15% oxygen vacancies were constructed. The electronic structures of pristine CeO_2_ and oxygen‐deficient CeO_2_ models were analyzed by calculating the PDOS and Bader charges. The NBANDS data were extracted from the OUTCAR file and multiplied by 2 for further analysis. The isosurface level of the differential charge density was set to 0.005 e Bohr^−3^.

The G point was utilized in the K‐point mesh, and the adsorption energy (*E*_ads_) was computed using the following formula:

(1) Eads=Etotal−Esubstrate−Eadsorbate

where *E*_total_、*E*_substrate_ and *E*_adsorbate_ represent the energies of the total structure, substrate, and adsorbate, respectively. The free energies were calculated using the following formula:

(2) G=EDFT+ZPE−TS

where *G*, *E*_DFT_, *ZPE*, and *TS* represent the free energy, energy from DFT calculations, zero‐point energy, and entropic contributions, respectively.

## Conflict of Interest

The authors declare no competing interests.

## Supporting information



Supporting Information

## Data Availability

Research data are not shared.
