# Supplementary material for: Valence‐Engineering of CeO2 Redox Modulator Boosts the Oxygen Electrocatalysis Performance in Fe/Co Dual‐Atom Catalyst
Source: Adv Sci (Weinh). 2025 Dec 20;13(11):e16405. doi: 10.1002/advs.202516405 (PMC12931224; doi:10.1002/advs.202516405)
Supplement: Supplementary file 1 — Supporting Information [file ADVS-13-e16405-s001.docx]

Supporting Information

Valence-engineering of CeO_2_ redox modulator boosts the oxygen electrocatalysis performance in Fe/Co dual-atom catalyst

Hengqi Liu ^a^, Jinzhen Huang ^b,f,*^, Shengyu Ma^a^, Rui Xiong^c^, Jiong Zhao^d^, Qiang Fu^a^，Hang Wei^e^，Zhiguo Liu^a,*^, Xianjie Wang^a^, Tai Yao^f^, and Bo Song^f, g, h, i, j, *^

^a^School of Physics, Harbin Institute of Technology, Harbin 150001, China

^b^Electrochemistry Laboratory, Paul Scherrer Institute, Villigen PSI, CH-5232, Switzerland

^c^School of future technology, Harbin Institute of Technology, Harbin 150001, China

^d^Department of Applied Physics, The Hong Kong Polytechnic University, Kowloon, Hong Kong 999077, China

^e^College of Chemistry and Chemical Engineering, Inner Mongolia Engineering and Technology Research Center for Catalytic Conversion and Utilization of Carbon Resource Molecules, Inner Mongolia University, Hohhot 010021, China

^f^National Key Laboratory of Science and Technology on Advanced Composites in Special Environments, Harbin Institute of Technology, Harbin 150001, China

^g^Laboratory for Space Environment and Physical Sciences, Harbin Institute of Technology, Harbin Institute of Technology, Harbin 150001, China

^h^National Key Laboratory of Laser Spatial Information, Harbin Institute of Technology, Harbin Institute of Technology, Harbin 150001, China

^i^Frontier Research Center of Space Environment Interacting with Matter, Harbin Institute of Technology, Harbin 150001, China

^j^Zhengzhou Research Institute, Harbin Institute of Technology, Zhengzhou, 450046, China

Correspondences should be addressed: Email: jinzhen.huang@hit.edu.cn;

[liuzhiguo@hit.edu.cn](mailto:liuzhiguo@hit.edu.cn); [songbo@hit.edu.cn](mailto:songbo@hit.edu.cn)

**Contents**

1. **Experimental Section** S-5
2. **Supplementary Figures and Tables**

Figure S1. The models of a) CeO_2_, b) CeO_2_-*V*_o_ (5%), c) CeO_2_-*V*_o_ (10%), and d) CeO_2_-*V*_o_ (15%). S-8

Figure S2. FeCo–N–C catalyst models (Fe and Co sites) and their preferred binding sites for ·OH, ·OOH, and g, h) H_2_O_2_ radicals S-9

Figure S3. CeO_2_ models with different oxygen vacancy (*V*_o_) concentrations and their preferred binding sites S-10

Figure S4. SEM images of ZIF-8, hollow ZIF-8, FeCo–N–C, and FeCo–N–C/CeO_2_-45

. S-11

Figure S5. TEM images of FeCo–N–C/CeO_2_-15, FeCo–N–C/CeO_2_-30 and FeCo–N–C/CeO_2_-60. S-12

Figure S6. HAADF-STEM image and c) SAED patterns of FeCo–N–C/CeO_2_-45. S-13

Figure S7. SEM and STEM images of CeO_2_. S-14

Figure S8. TEM image and energy-dispersive X-ray spectroscopy maps S-15

Figure S9. XRD patterns of ZIF-8, ZIF-8 (hollow) and CeO_2_, FeCo–N–C and FeCo–N–C/CeO_2_.

S-16

Figure S10. Determination of the Ce^3+^/Ce^4+^ ratio in the catalysts by chemical titration.

S-17

Figure S11. XPS survey spectra of FeCo–N–C, FeCo–N–C/CeO_2_, FeCo–N–C/CeO_2_-15, FeCo–N–C/CeO_2_-30, FeCo–N–C/CeO_2_-45, and FeCo–N–C/CeO_2_-60. S-19

Figure S12. The C 1*s* and N 1*s* XPS spectra of the samples S-20

Figure S13. The O 1*s* and Ce 3*d* XPS spectra of the samples S-21

Figure S14. Co and Fe K-edge XANES and FT-EXAFS spectra S-22

Figure S15. Raman spectra of of FeCo–N–C, FeCo–N–C/CeO_2_, FeCo–N–C/CeO_2_-15, FeCo–N–C/CeO_2_-30, FeCo–N–C/CeO_2_-45, and FeCo–N–C/CeO_2_-60. S-23

Figure S16. N_2_ adsorption-desorption isotherms and pore size distribution S-24

Figure S17. Cyclic voltammetry (CV) curves of the catalysts collected in O_2_-saturated 0.1 M KOH electrolyte at a scan rate of 10 mV s^‑1^. S-25

Figure S18. Linear sweep voltammetry (LSV) curves of the catalysts collected at rotating rates ranging from 100 to 1600 rpm. S-26

Figure S19. LSV curves, Tafel plots, The electron transfer number and H_2_O_2_ yield

S-27

Figure S20. The i-t curves of FeCo–N–C/CeO_2_-15, FeCo–N–C/CeO_2_-30 and FeCo–N–C/CeO_2_-60 in O_2_-saturated 0.1 M KOH electrolyte at 0.75 V vs. RHE. S-28

Figure S21. OER LSV curves of in 1.0 M KOH and their corresponding Tafel slopes.

S-29

Figure S22. CV curves collected in the potential window of 1.024-1.124 V vs. RHE at various scan rates S-30

Figure S23. CV curves collected in the potential window of 1.024-1.124 V vs. RHE at various scan rates S-31

Figure S24. The current density difference at 1.074 V vs. RHE is plotted as a function of scan rate to extract the *C*_dl_ S-32

Figure S25. The i-t curves of a) FeCo–N–C, FeCo–N–C/CeO_2_, b) FeCo–N–C/CeO_2_-15, FeCo–N–C/CeO_2_-30, and FeCo–N–C/CeO_2_-60 recorded at 1.58 V vs. RHE. S-33

Figure S26. Fluorescence spectra of coumarin dye after reaction with radicals generated by the Fenton reaction system S-34

Figure S27. The CV curves of the FeCo–N–C recorded in KOH solutions of different pH values, and the magnified CV curves of FeCo–N–C at different scan rates in 1.0 M KOH solution S-35

Figure S28. Nyquist plots for FeCo–N–C and FeCo–N–C/CeO_2_ during the OER process. S-36

Figure S29. Free energies of the intermediates for the OER (left to right) and ORR (right to left) in the FeCo–N–C (Co_0.72_Fe_0.28_) and FeCo–N–C (FeCo-N_6_) catalysts. S-37

Figure S30. The OER reaction processes S-38

Figure S31. Band gap spectra of a) CeO_2_, b) CeO_2_-15, c) CeO_2_-30, d) CeO_2_-45, and e) CeO_2_-60. S-39

Figure S32. Galvanostatic charge/discharge cycling curves of the FeCo–N–C/CeO_2_-45 catalyst at 10 mA cm^-2^ and the corresponding round-trip efficiency................................ S-40

Figure S33. SEM images of the zinc anode a) before and b) after cycling in the zinc–air battery................................ S-41

Figure S34. The Ce 3*d* spectra of FeCo–N–C/CeO_2_-45 after electrochemical testing and b) the variation trends of Ce^3+^ and Ce^4+^ after electrochemical testing. S-42

Table S1. The percentages (%) of different N species in the samples obtained by XPS analysis S-43

Table S2. Comparison of the ZABs performance of FeCo–N–C/CeO_2_-45 to another related bifunctional catalysts in literature S-44

**3. References in Supplementary Information** S-45

**1.Experimental Section**

**Materials**

2-Methylimidazole, cerium nitrate hexahydrate (Ce(NO_3_)_3_·6H_2_O), zinc nitrate hexahydrate (Zn(NO_3_)_2_·6H_2_O), cobalt nitrate hexahydrate (Co(NO_3_)_2_·6H_2_O), ferric nitrate nonahydrate (Fe(NO_3_)_3_·9H_2_O), sodium borohydride, n-hexane, standard 20 % Pt/C, and 99.5 % RuCl_3_ catalysts were purchased from Aladdin. Tannic acid was obtained from Tianjin Damao Chemical Reagent Co., Ltd. All reagents were used as received without further purification.

**Preparation of CeO_2_ NPs.**

First, 3.5 mmol Ce(NO_3_)_3_·6H_2_O was dissolved in 40 mL deionized water. Ammonia solution was added dropwise to adjust the pH of the solution to 10. The solution was subsequently transferred to a reactor, and subjected to a hydrothermal reaction at 120 °C for 5 h. After the reaction, the heating source was turned off immediately. The obtained sample was washed with ethanol and deionized water, followed by centrifugation to collect the precipitate. The product was then dried at 65 °C. The resulting sample was separately placed in 1 mol/L NaBH_4_ solution for etching at 15, 30, 45, and 60 min, respectively. Afterwards, the samples were thoroughly washed with anhydrous ethanol and deionized water, then dried to obtain CeO_2_ NPs with different valence state ratios, denoted as CeO_2_-15, CeO_2_-30, CeO_2_-45, and CeO_2_-60.

**Physicochemical characterization**

The crystal structure and chemical composition of the samples were analyzed using a Rigaku D/max 2500 X-ray diffraction analyzer (XRD) and an ESCALAB250 X-ray photoelectron spectrometer (XPS) with an Al Kα source, respectively. The morphology and microstructure were examined using a Hitachi SU8020 scanning electron microscope (SEM) and an FEI Talos F200X transmission electron microscope (TEM). Aberration-Corrected High-Angle Annular Dark Field Scanning Transmission Electron Microscopy (AC-HAADF-STEM) characterization was performed using a ARM300 microscope operated at 200 kV. The Raman spectra were collected using an HR 800 Raman spectrometer with a 633 nm laser. The Brunauer-Emmett-Teller (BET) specific surface areas were determined by conducting the nitrogen adsorption-desorption measurements using an ASAP 2460. X-ray absorption spectroscopy (XAS) spectra were collected at the RapidXAFS and analyzed with the Demeter program package (Version 0.9.26).

**Electrochemical measurements**

The ORR measurements were carried out in a three-electrode system configuration using a CHI760E electrochemical workstation (Shanghai Chenhua). An RRDE (glassy C as the disk with an area of *A* = 0.19625 cm^2^ and Pt as the ring with an area of *A* = 0.2198 cm^2^) was used as the substrate for preparing the working electrodes. The sample inks were prepared by adding 2.5 mg of catalyst in a 1000-μL solution containing 485 μL of deionized water, 485 μL of isopropanol (C_3_H_8_O), and 30 μL of Nafion (5 wt.%). The resulting solution was ultrasonicated until a homogeneous suspension was obtained. Next, 8 μL of the suspension drop-cast was dropped onto the glassy C disk. Hg/HgO and a graphite rod for the ORR (a Pt foil for the OER) were used as the reference and counter electrodes, respectively.

The CV curves were recorded in the voltage range from 0.2 to 1.0 V vs. Hg/HgO at a scan rate of 50 mV s^−1^ in the N_2_/O_2_-saturated 0.1-M KOH. The LSV curves were recorded in the same potential range at various rotation speeds (100, 400, 900, 1600, and 2500 rpm) at a scan rate of 10 mV s^−1^, with the ring being constantly polarized at 1.3 V. vs. RHE The potentials were estimated according to the following equation:

$\text{E}_{\text{RHE}}\text{ = }\text{E}_{\text{0}}\text{ + }\text{E}_{\text{Hg/HgO}}\text{ + 0.059 × }\text{pH}\text{ - }\text{I}_{\text{d}}\text{R}_{\text{s}}$,  (S1)

where *E*_0_ represents the measured potential, *E_Hg/HgO_* = 0.098 V, *I_d_* denotes the disk current, and *R_s_* is the resistance of the electrolyte.

The value of *n* was also determined by calculating the H_2_O_2_ yield derived from the ring current. The H_2_O_2_ yield and *n* were calculated according to the following equations:

$\text{\%}\text{H}_{\text{2}}\text{O}_{\text{2}}\text{ = 200}\frac{\frac{\text{i}_{\text{r}}}{\text{N}}}{\text{i}_{\text{d}}\text{ + }\frac{\text{i}_{\text{r}}}{\text{N}}}\text{ = 200}\frac{\text{i}_{\text{r}}}{\text{N}\text{i}_{\text{d}}\text{ + }\text{i}_{\text{r}}}$, (S2)

$\text{n }\text{= 4}\frac{\text{i}_{\text{d}}}{\text{i}_{\text{d}}\text{ + }\frac{\text{i}_{\text{r}}}{\text{N}}}\text{ = 4}\frac{\text{N}\text{i}_{\text{d}}}{\text{N}\text{i}_{\text{d}}\text{ + }\text{i}_{\text{r}}}$, (S3)

where *i_r_* and *N* are the ring current and the collection efficiency of the Pt ring (0.424), respectively.

For the OER measurements, CV experiments were conducted to reach a stable state within an O_2_-saturated 1 M aqueous KOH solution at a scan rate of 50 mV s^−1^. The LSV curves were recorded at a scan rate of 10 mV s^−1^ and analyzed by applying a 95% *iR* correction. Operando electrochemical impedance spectroscopy (EIS) was performed over a frequency range of 100 kHz to 0.1 Hz to evaluate the interfacial charge transfer characteristics of the catalyst at different potentials.

**ZABs assembling and testing**

The rechargeable ZAB was mainly composed of a polished zinc plate with a thickness of 0.5 mm, 6.0-M KOH with 0.2-M Zn(CH_3_COO)_2_, and a carbon paper/nickel foam mixed substrate loaded with a catalyst with a density of 1 mg cm^−2^, which acted as the anode, electrolyte, and air cathode, respectively. A mixed catalyst containing commercial Pt/C and RuO_2_ with a mass ratio of 1:1 was also prepared for comparison. All the electrochemical measurements were performed using an electrochemical workstation (CHI 760E) at room temperature and under ambient air conditions. All measurements were conducted using the same polished zinc foil and the same batch of electrolyte.

**Fluorescence Spectroscopy for Radical Scavenging Evaluation**

Firstly, ·OH radicals are generated through the Fenton reaction (Fe^2+^/H_2_O_2_ system). The prepared FeCo–N–C, FeCo–N–C/CeO_2_, and FeCo–N–C/CeO_2_-45 catalysts (1 mg) are ultrasonically dispersed in 1 mL 0.1 M H_2_SO_4_ solution to form uniform suspension, respectively. Then, 200 μL (1 mg/mL) of catalyst solution and varying amounts of H₂O₂ are added to 5 mL of coumarin solution (0.5 mM, dissolved in 0.1 M H_2_SO_4_). The non-fluorescent coumarin dye is used as a fluorescent molecular probe, as it is sensitive to free radicals and generates strong fluorescence of 7-hydroxycoumarin upon free radical attack. Fluorescence changes of coumarin (relative to the initial intensity) are measured using a fluorescence spectrophotometer at time intervals of 10, 30, 60, 90, and 120 min.

**2.Supplementary Figures and Tables**


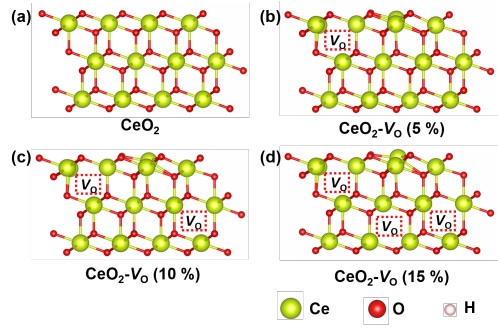


**Figure S1.** The models of a) CeO_2_, b) CeO_2_-*V*_O_ (5%), c) CeO_2_-*V*_O_ (10%), and d) CeO_2_-*V*_O_ (15%).


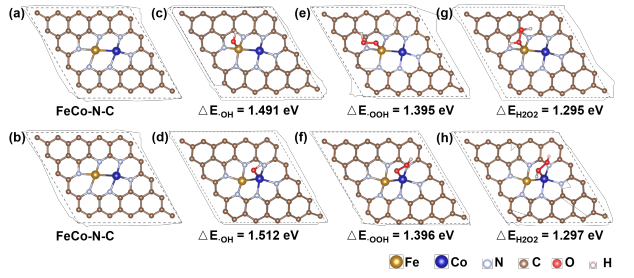


**Figure S2.** a, b) Top-down views of the FeCo–N–C catalyst models (Fe and Co sites) and their preferred binding sites for c, d) ·OH, e, f) ·OOH, and g, h) H_2_O_2_ radicals, along with the calculated adsorption energies. The orange, blue, white, brown, red, and pink spheres represent Fe, Co, N, C, O, and H atoms, respectively.


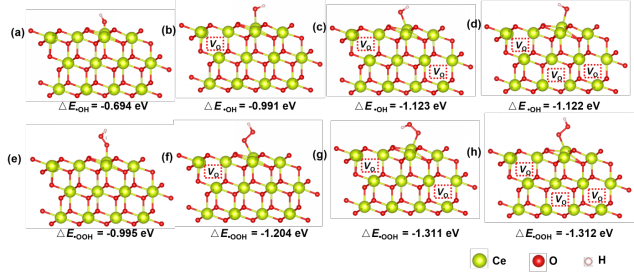


**Figure S3.** CeO_2_ models with different *V*_O_ concentrations and their preferred binding sites for a-d) ·OH and e-h) ·OOH, along with the calculated adsorption energies. The yellow, red, and pink spheres represent Ce, O, and H atoms, respectively.


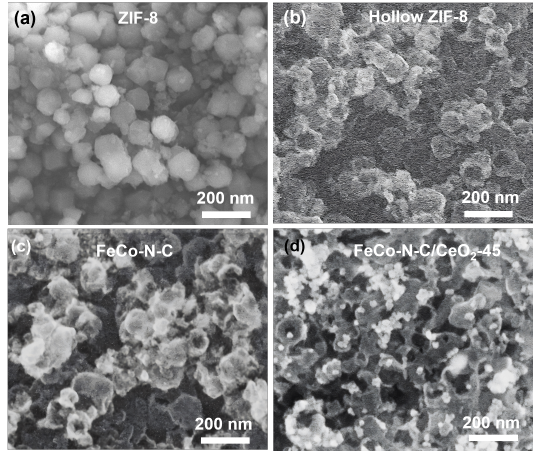


**Figure S4.** SEM images of a) ZIF-8, b) hollow ZIF-8, c) FeCo–N–C, and d) FeCo–N–C/CeO_2_-45.


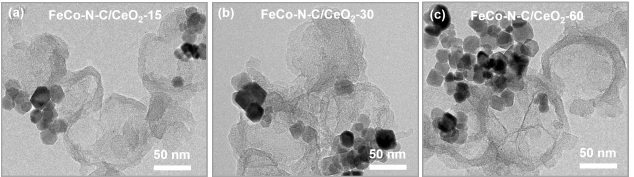


**Figure S5.** TEM images of a) FeCo–N–C/CeO_2_-15, b) FeCo–N–C/CeO_2_-30 and c) FeCo–N–C/CeO_2_-60.


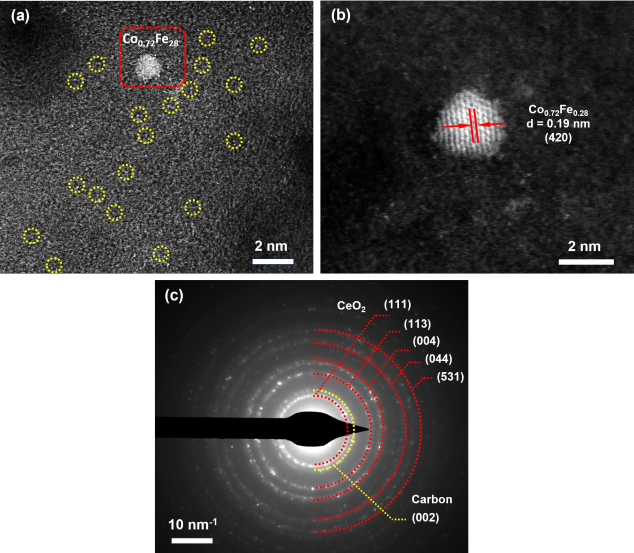


**Figure S6.** a, b) HAADF-STEM image and c) SAED patterns of FeCo–N–C/CeO_2_-45.


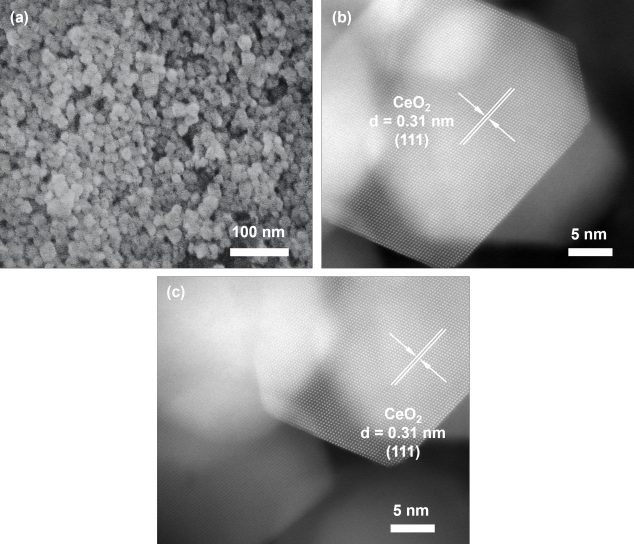


**Figure S7.** a) SEM images of CeO_2_. b, c) STEM images of CeO_2_.


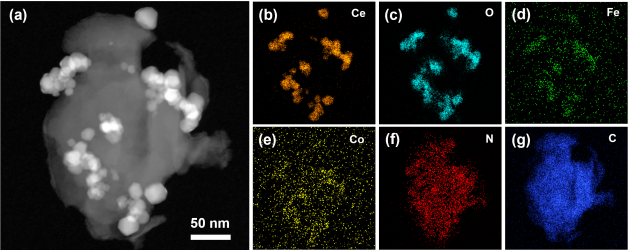


**Figure S8.** a) TEM image and energy-dispersive X-ray spectroscopy maps of b) Ce, c) O, d) Fe, e) Co, f) N, and g) C elements in FeCo–N–C/CeO_2_-45.


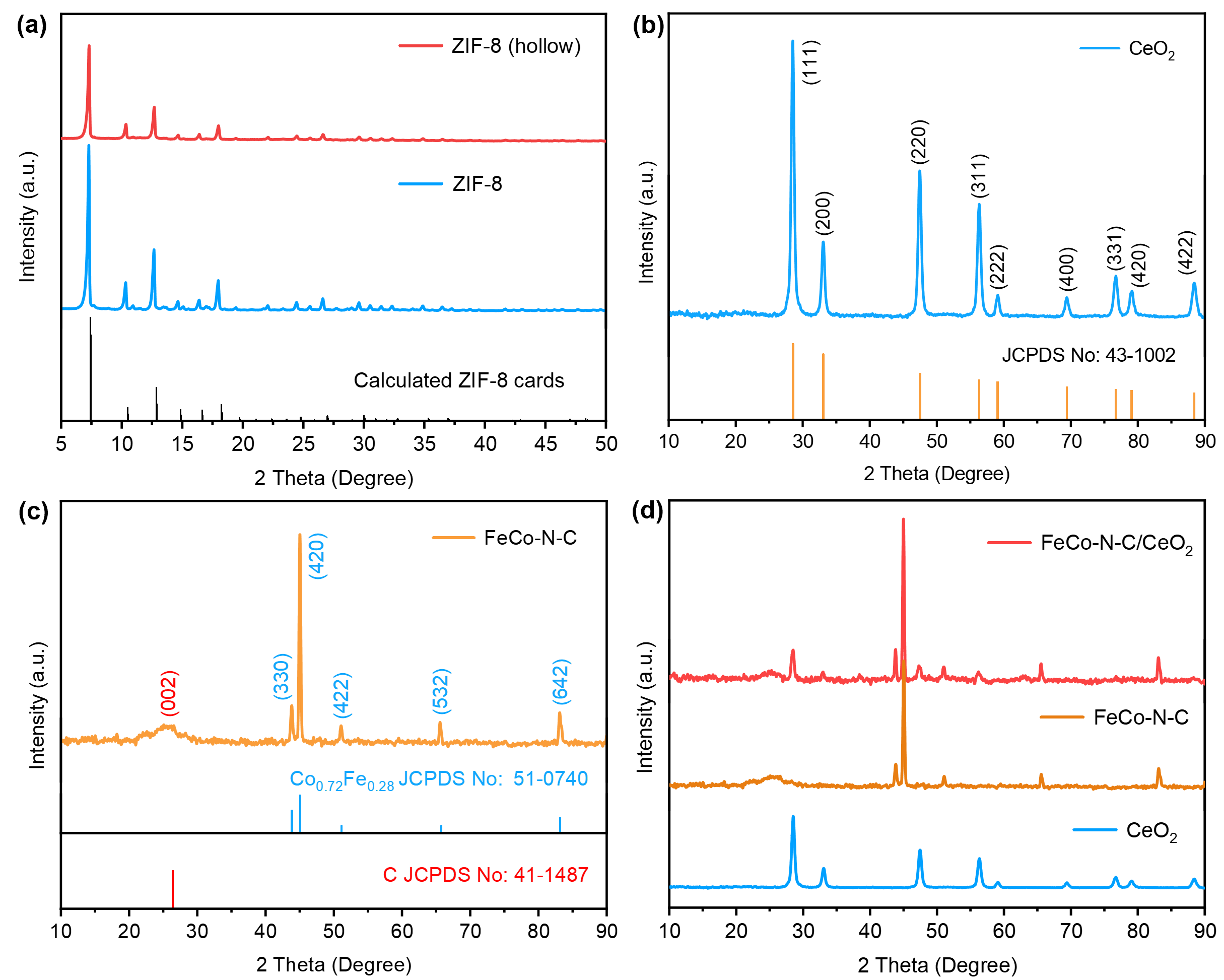


**Figure S9.** XRD patterns of a) ZIF-8 and ZIF-8 (hollow), b) CeO_2_, c) FeCo–N–C and d) CeO_2_, FeCo–N–C and FeCo–N–C/CeO_2_.


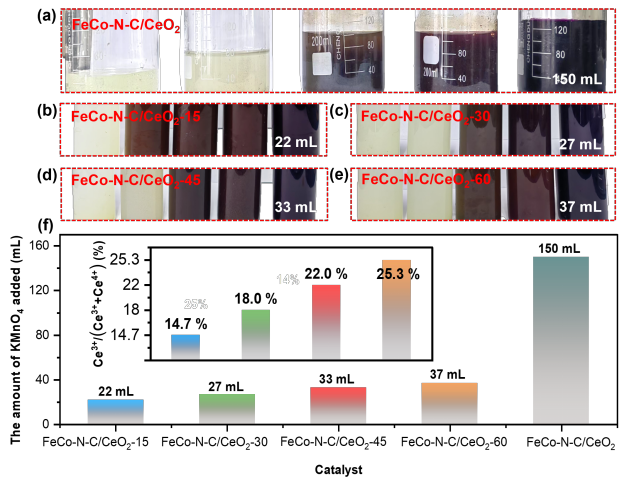


**Figure S10.** Determination of the Ce^3+^/Ce^4+^ ratio in the catalysts by chemical titration. During the experiment, a standard solution was added dropwise to the sample solution using a burette, and the endpoint is identified by the color change of the KMnO_4_ indicator. The molar amount of Ce^3+^ in the sample is calculated based on the volume of 0.02 mol/L KMnO_4_ solution consumed for the titration. Photos showing the color changes during the titration process for a) FeCo–N–C/CeO_2_, b) FeCo–N–C/CeO_2_-15, c) FeCo–N–C/CeO_2_-30, d) FeCo–N–C/CeO_2_-45, and e) FeCo–N–C/CeO_2_-60. f) Statistical results.

Discussions of chemical titration

In acidic conditions, Ce^3+^ ions reacts with potassium permanganate (KMnO_4_) in a redox reaction, as shown below:

$\text{5}\text{Ce}^{\text{3+}}\text{ + }\text{MnO}_{\text{4}}^{\text{-}}\text{ + 8 }\text{H}^{\text{+}}\text{ → }\text{5}\text{Ce}^{\text{4+}}\text{+ }\text{MnO}^{\text{2+}}\text{ + 4}\text{H}_{\text{2}}\text{O}$ (S4)

Ce^3+^ ions are oxidized to Ce^4+^ ions, while KMnO_4_ is reduced to Mn^2+^. During the reaction, the purple color of KMnO_4_ fades. Since Ce^4+^ ions do not react with KMnO_4_, the amount of KMnO_4_ consumed during titration can be used to calculate the proportion of Ce^3+^.

Reference sample (FeCo–N–C/CeO_2_): 150 mL of KMnO_4_ corresponds to 100% Ce^3+^. KMnO_4_ volumes consumed for other samples: 22 mL (FeCo–N–C/CeO_2_-15), 27 mL (FeCo–N–C/CeO_2_-30), 33 mL (FeCo–N–C/CeO_2_-45), and 38 mL (FeCo–N–C/CeO_2_-60).

The proportion of Ce^3+^ in the total Ce content is calculated using the following formula:

$\text{Ce}^{\text{3+}}\text{proportion =}\frac{\text{Volume of }\text{KMnO}_{\text{4}}\text{​}\text{ consumed by the samples}}{\text{Volume of }\text{KMnO}_{\text{4}}\text{​}\text{ consumed by the reference sample}}\text{ ×100 \%}$ (S5)

The calculations for each sample are as follows:

FeCo–N–C/CeO_2_-15: Ce^3+^ proportion = 14.7%,

FeCo–N–C/CeO_2_-30: Ce^3+^ proportion = 18.0%,

FeCo–N–C/CeO_2_-45: Ce^3+^ proportion = 22.0%,

FeCo–N–C/CeO_2_-60: Ce^3+^ proportion = 25.3%.


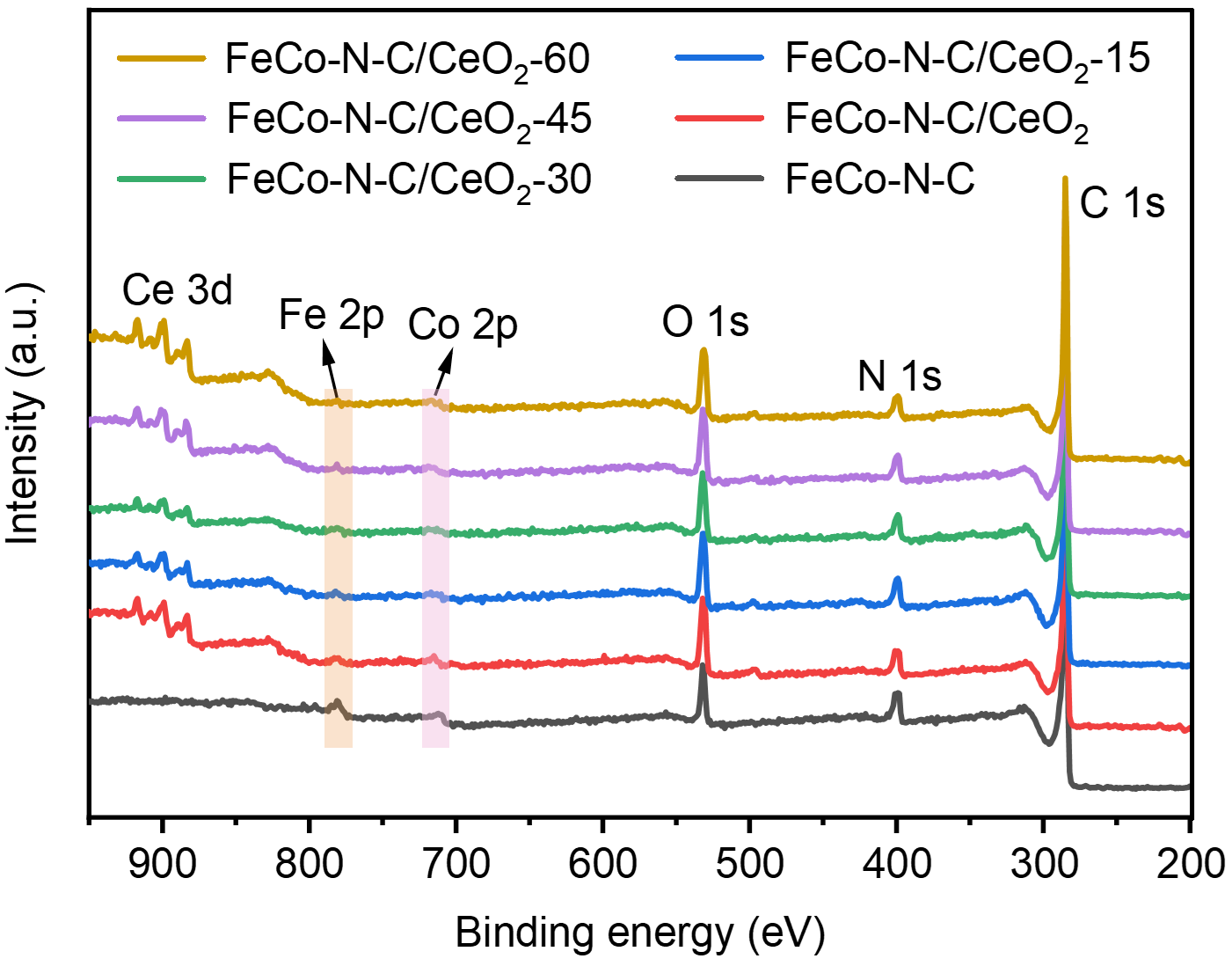


**Figure S11.** XPS survey spectra of FeCo–N–C, FeCo–N–C/CeO_2_, FeCo–N–C/CeO_2_-15, FeCo–N–C/CeO_2_-30, FeCo–N–C/CeO_2_-45, and FeCo–N–C/CeO_2_-60.


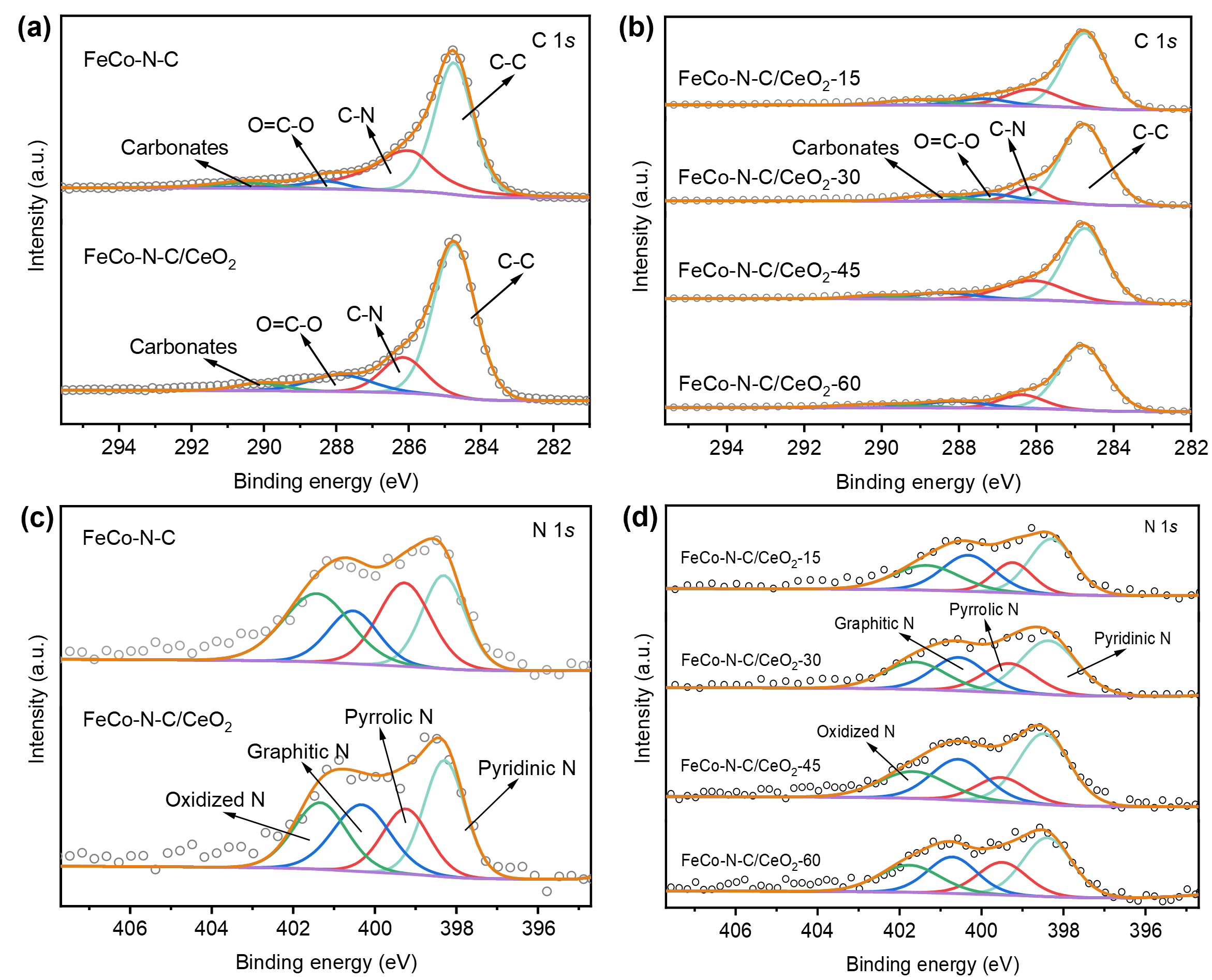


**Figure S12.** The C 1*s* XPS spectra of a) FeCo–N–C, FeCo–N–C/CeO_2_, b) FeCo–N–C/CeO_2_-15, FeCo–N–C/CeO_2_-30, FeCo–N–C/CeO_2_-45, and FeCo–N–C/CeO_2_-60. The N 1*s* XPS spectra of c) FeCo–N–C, FeCo–N–C/CeO_2_, d) FeCo–N–C/CeO_2_-15, FeCo–N–C/CeO_2_-30, FeCo–N–C/CeO_2_-45, and FeCo–N–C/CeO_2_-60.


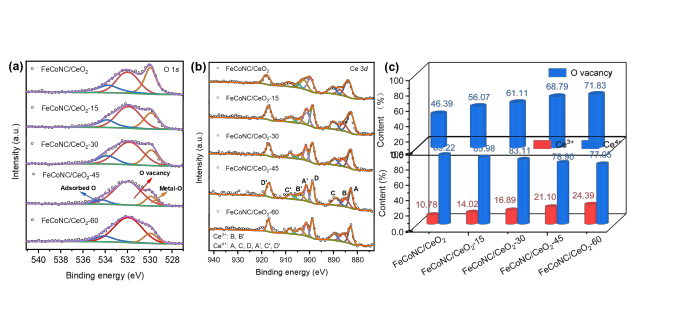


**Figure S13.** a) The Ce 3*d* XPS spectra of FeCo–N–C/CeO_2_, FeCo–N–C/CeO_2_-15, FeCo–N–C/CeO_2_-30, FeCo–N–C/CeO_2_-45, and FeCo–N–C/CeO_2_-60. b) The O 1*s* XPS spectra of FeCo–N–C/CeO_2_, FeCo–N–C/CeO_2_-15, FeCo–N–C/CeO_2_-30, FeCo–N–C/CeO_2_-45, and FeCo–N–C/CeO_2_-60. c) The variation trends of Ce^3+^, Ce^4+^, and O_v_ contents with increasing immersion time in NaBH_4_ solution.


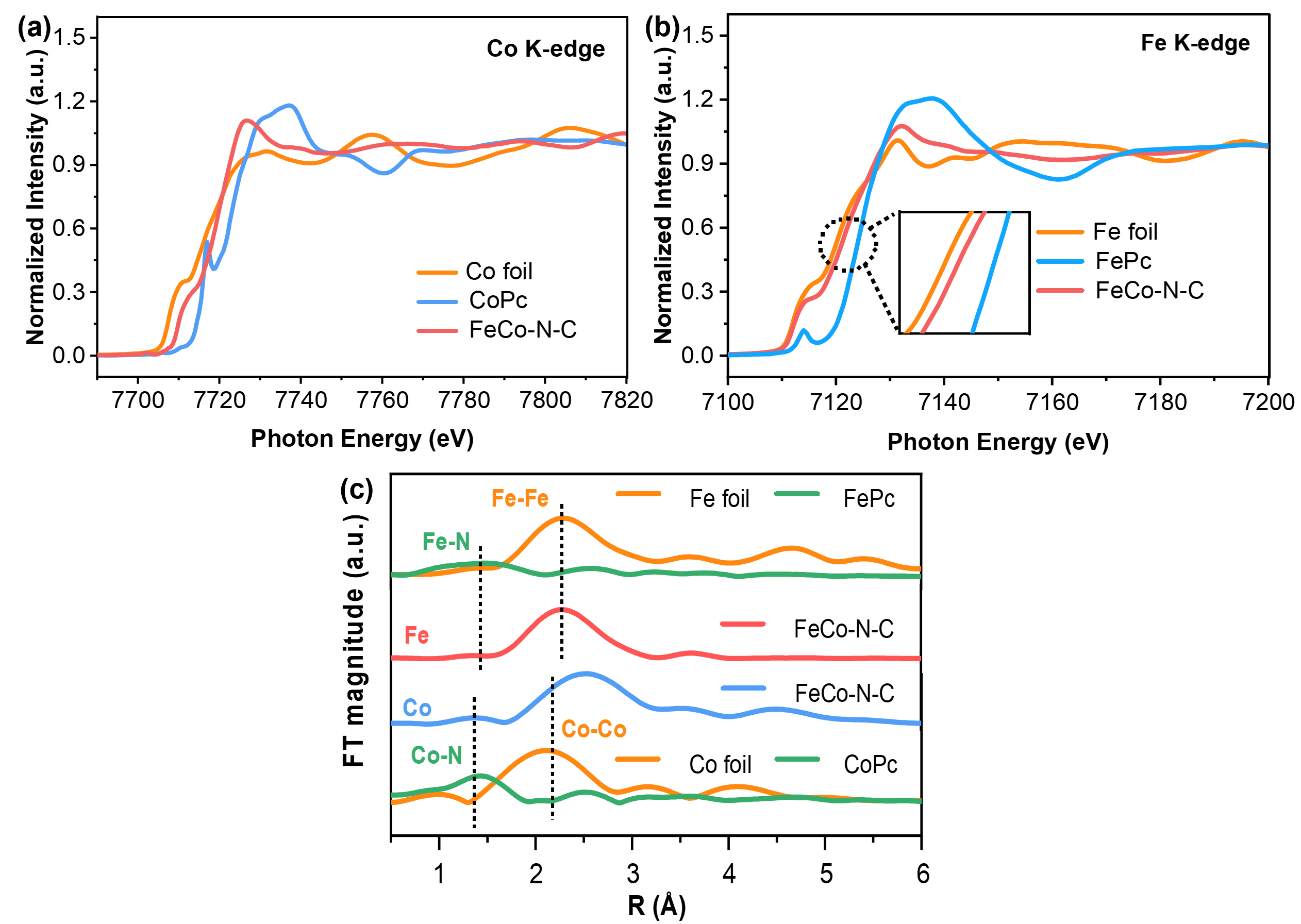


**Figure S14.** a) Co K-edge XANES spectra of Co foil, CoPc, and FeCo–N–C. b) Fe K-edge XANES spectra of Fe foil, FePc, and FeCo–N–C. c) FT-EXAFS spectra of Fe foil, FePc, Co foil, CoPc, and FeCo–N–C.


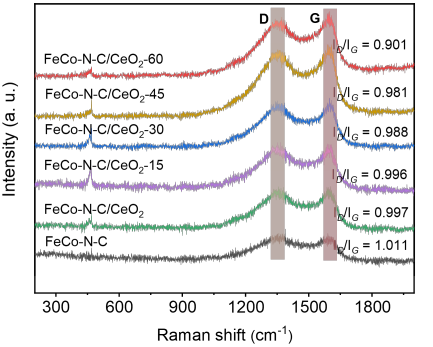


**Figure S15.** Raman spectra of of FeCo–N–C, FeCo–N–C/CeO_2_, FeCo–N–C/CeO_2_-15, FeCo–N–C/CeO_2_-30, FeCo–N–C/CeO_2_-45, and FeCo–N–C/CeO_2_-60.


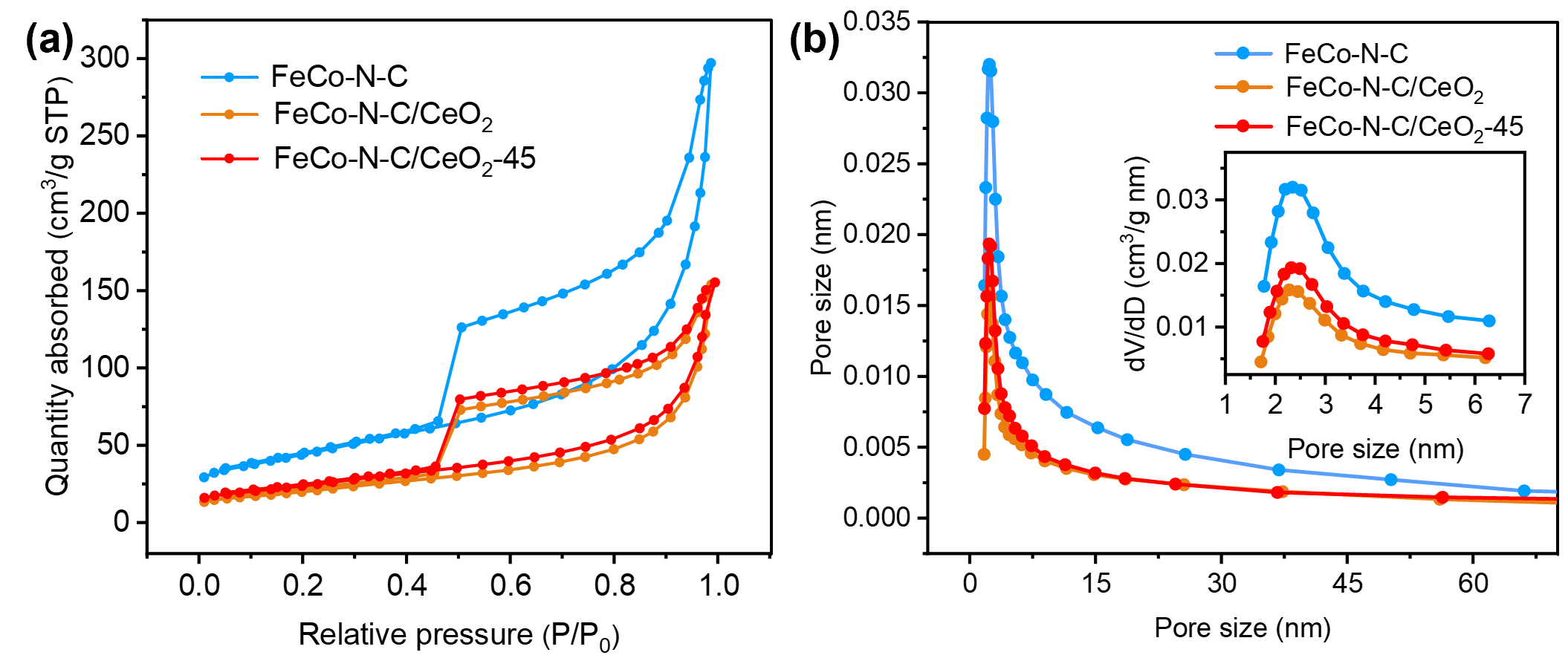


**Figure S16.** a) N_2_ adsorption-desorption isotherms and b) pore size distribution of FeCo–N–C, FeCo–N–C/CeO_2_, FeCo–N–C/CeO_2_-45, The inset in panel b) is the enlarged figure to show the features at small pore size range.


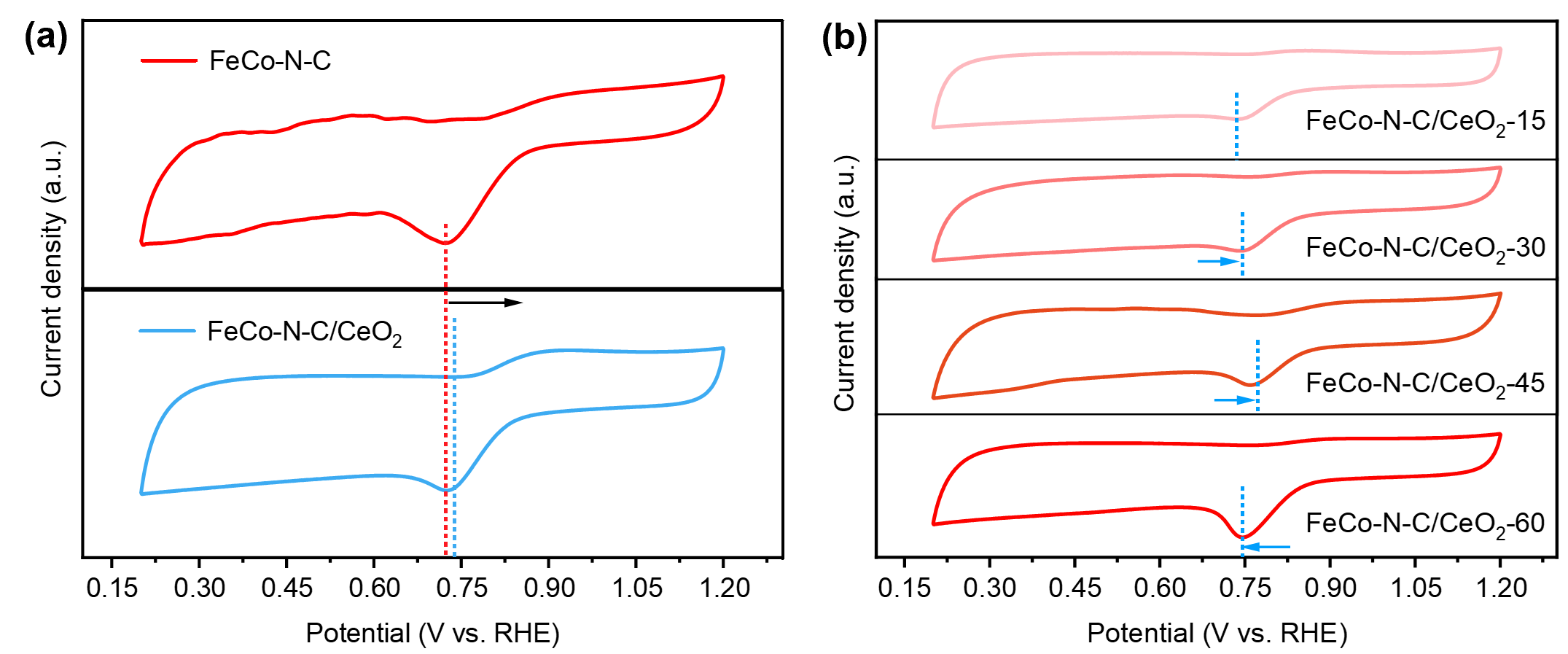


**Figure S17.** Cyclic voltammetry (CV) curves of a) FeCo–N–C, FeCo–N–C/CeO_2_, b) FeCo–N–C/CeO_2_-15, FeCo–N–C/CeO_2_-30, FeCo–N–C/CeO_2_-45 and FeCo–N–C/CeO_2_-60 collected in O_2_-saturated 0.1 M KOH electrolyte at a scan rate of 10 mV s^‑1^.


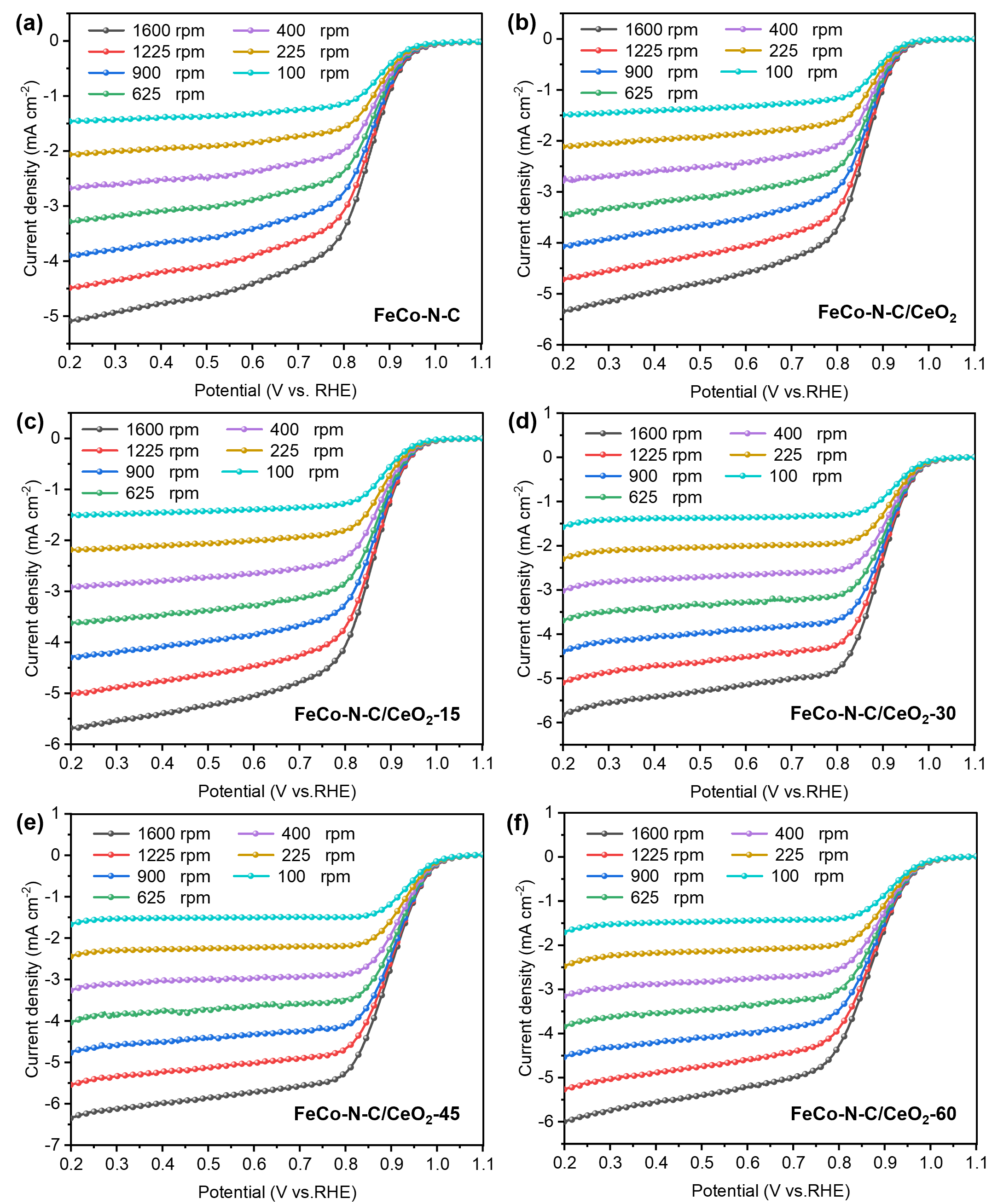


**Figure S18.** Linear sweep voltammetry (LSV) curves of a) FeCo–N–C, b) FeCo–N–C/CeO_2_, c) FeCo–N–C/CeO_2_-15, d) FeCo–N–C/CeO_2_-30, e) FeCo–N–C/CeO_2_-45 and f) FeCo–N–C/CeO_2_-60 collected at rotating rates ranging from 100 to 1600 rpm.


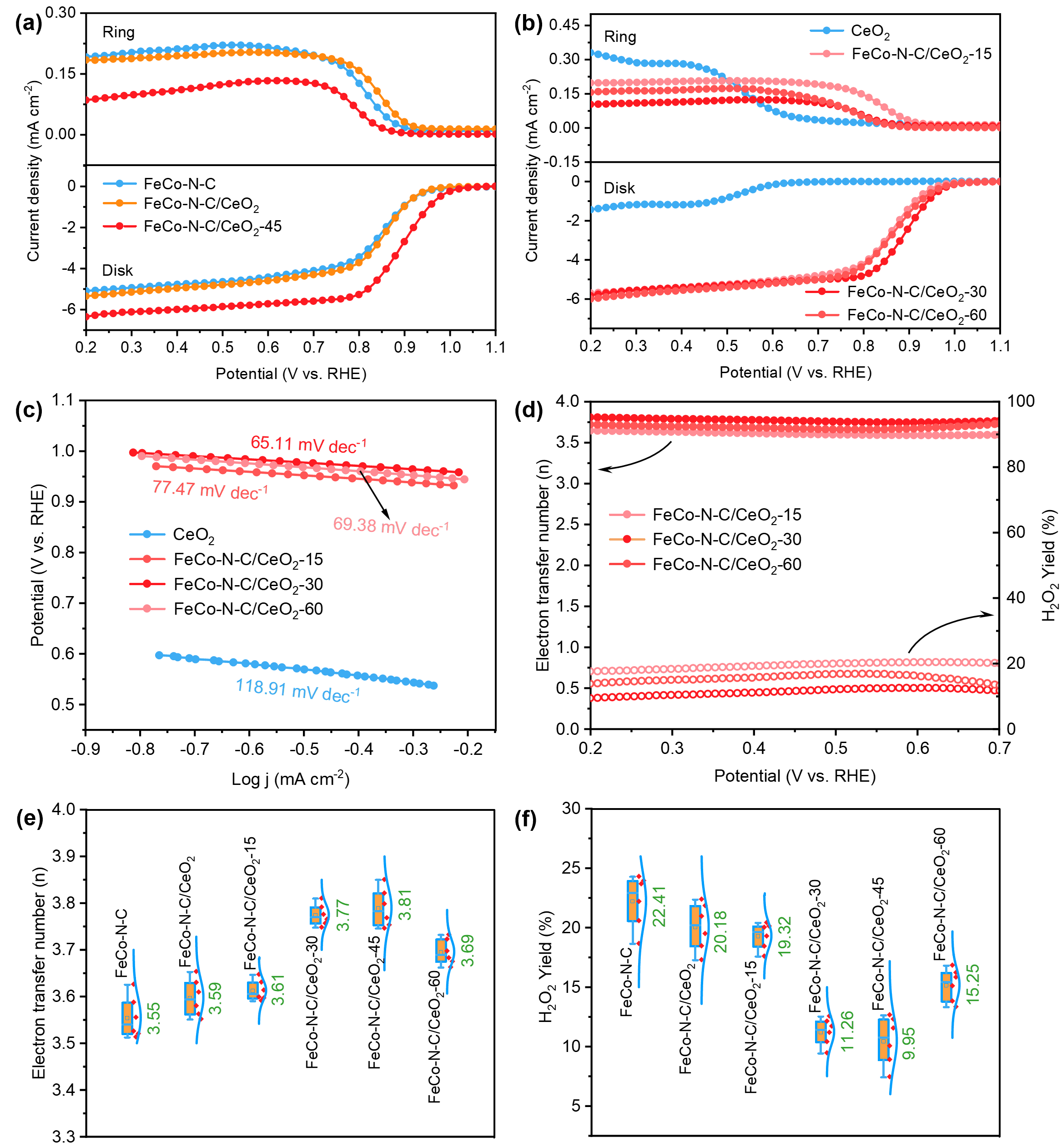


**Figure S19.** a) LSV curves of FeCo–N–C, FeCo–N–C/CeO_2_, FeCo–N–C/CeO_2_-45, b) CeO_2_, FeCo–N–C/CeO_2_-15, FeCo–N–C/CeO_2_-30 and FeCo–N–C/CeO_2_-60 recorded at the glassy carbon (disk) and Pt (ring) electrode of RRDE at a scan rate of 10 mV s^-1^ and a rotating speed of 1600 rpm. c) Tafel plots of CeO_2_, FeCo–N–C/CeO_2_-15, FeCo–N–C/CeO_2_-30 and FeCo–N–C/CeO_2_-60. d) The electron transfer number and H_2_O_2_ yield calculated from rotation dependent LSV curves of the above catalysts at voltages ranging from 0.2 to 0.7 V (Figure S15b). The normal distribution statistics of e) the electron transfer number and f) H_2_O_2_ yield of the catalysts at the voltages ranging from 0.2 to 0.7 V.


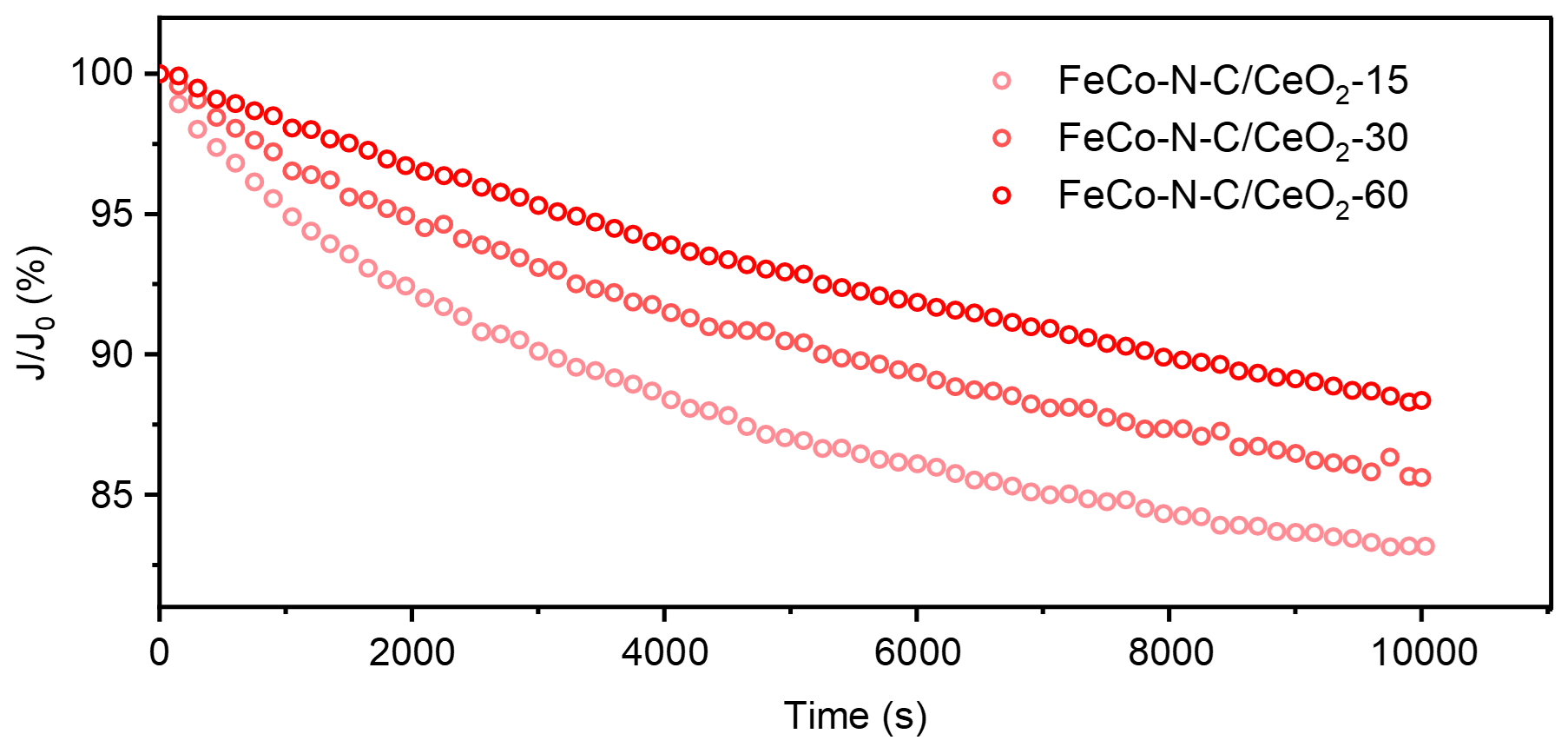


**Figure S20.** The i-t curves of FeCo–N–C/CeO_2_-15, FeCo–N–C/CeO_2_-30 and FeCo–N–C/CeO_2_-60 in O_2_-saturated 0.1 M KOH electrolyte at 0.75 V vs. RHE.


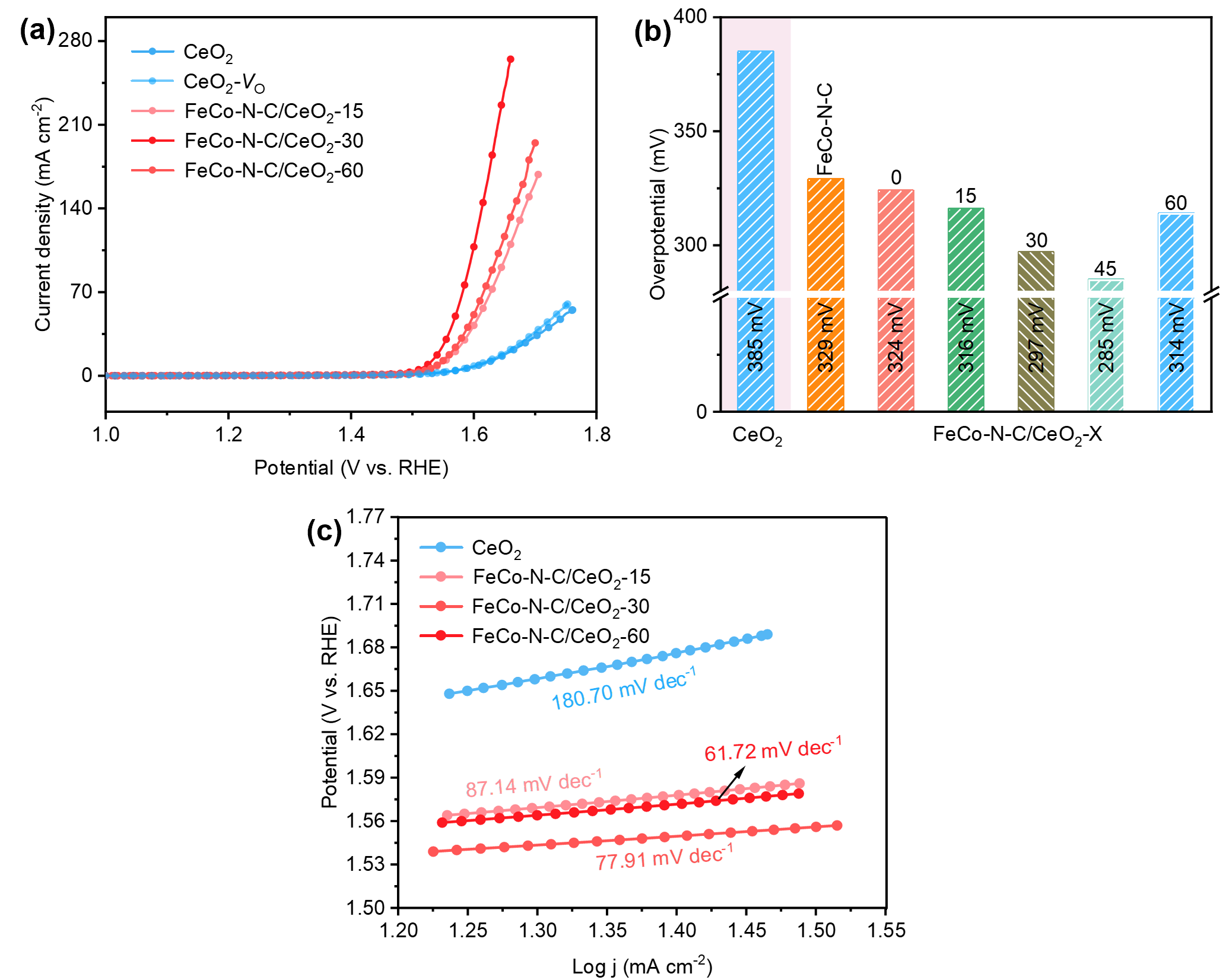


**Figure S21.** a) LSV curves of CeO_2_, CeO_2_-*V*_O_, FeCo–N–C/CeO_2_-15, FeCo–N–C/CeO_2_-30 and FeCo–N–C/CeO_2_-60, to compare the OER performances in 1.0 M KOH. b) The comparison of OER overpotential for the above samples and c) their corresponding Tafel slopes.


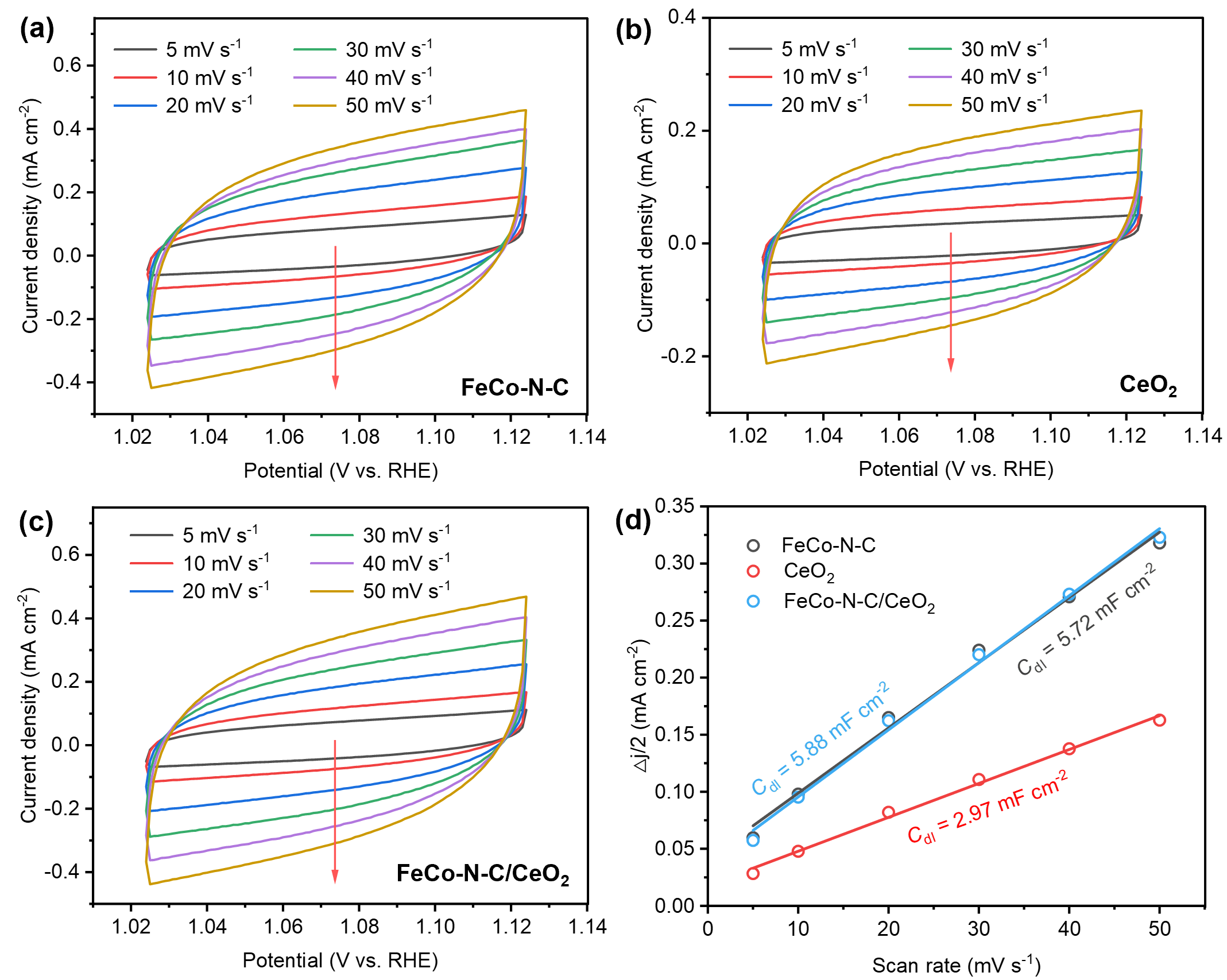


**Figure S22.** CV curves collected in the potential window of 1.024-1.124 V vs. RHE at various scan rates for a) FeCo–N–C, b) CeO_2_ and c) FeCo–N–C/CeO_2_. d) The current density difference at 1.074 V vs. RHE is plotted as a function of scan rate to extract the double layer capacitance (*C*_dl_).


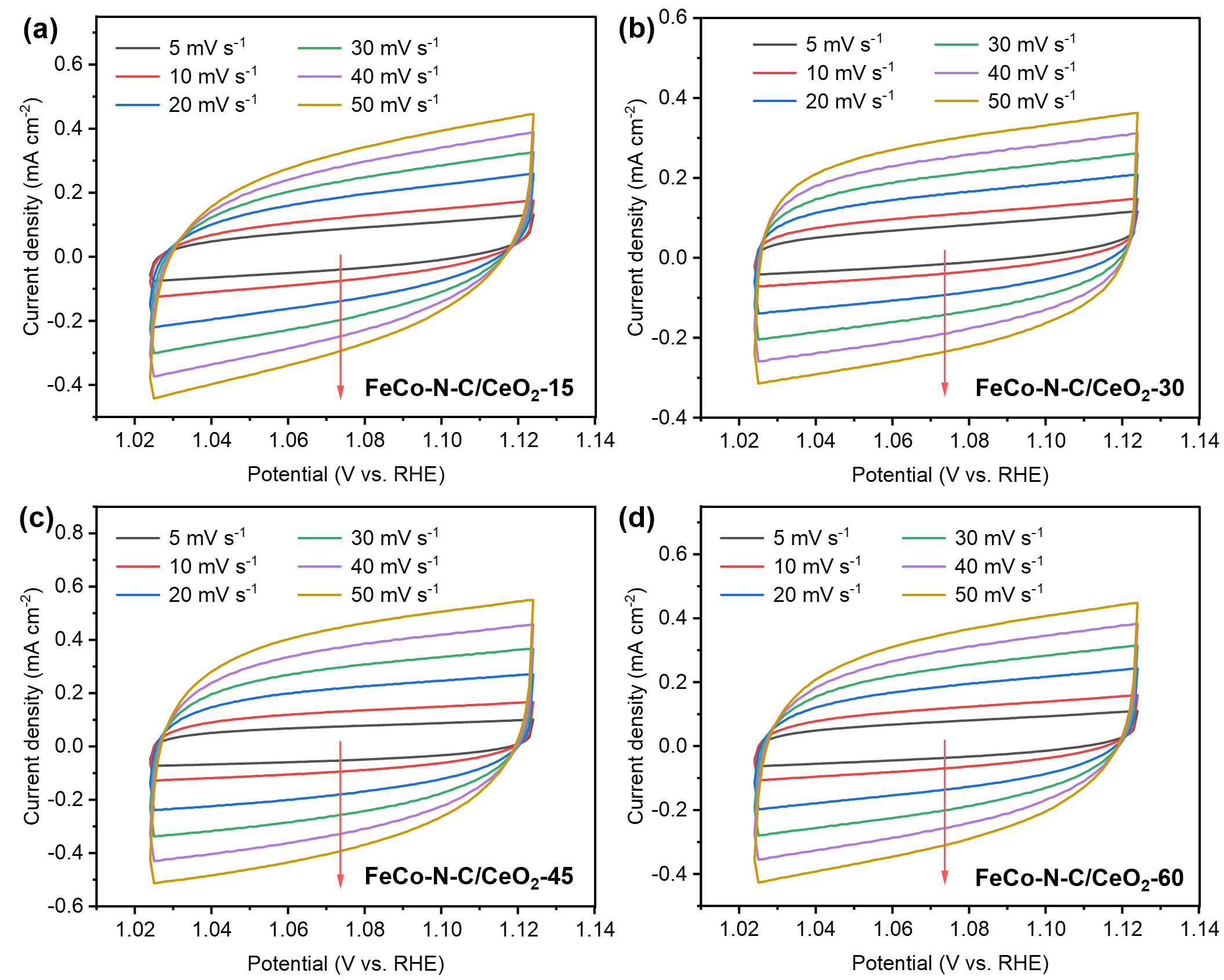


**Figure S23.** CV curves collected in the potential window of 1.024-1.124 V vs. RHE at various scan rates for a) FeCo–N–C/CeO_2_-15, b) FeCo–N–C/CeO_2_-30, c) FeCo–N–C/CeO_2_-45, and d) FeCo–N–C/CeO_2_-60.


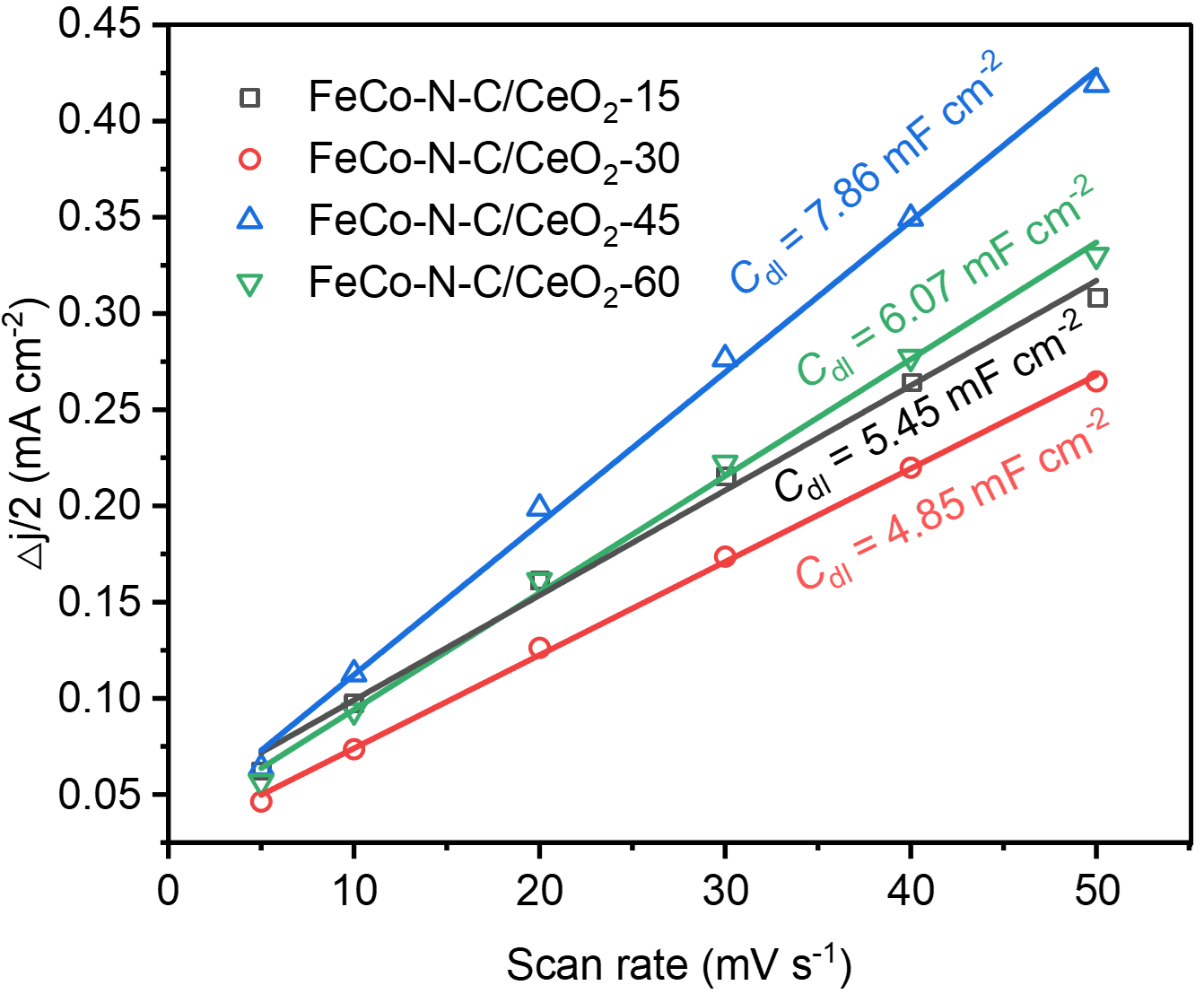


**Figure S24.** The current density difference at 1.074 V vs. RHE is plotted as a function of scan rate to extract the *C*_dl_ for FeCo–N–C/CeO_2_-15, FeCo–N–C/CeO_2_-30, FeCo–N–C/CeO_2_-45, and FeCo–N–C/CeO_2_-60.


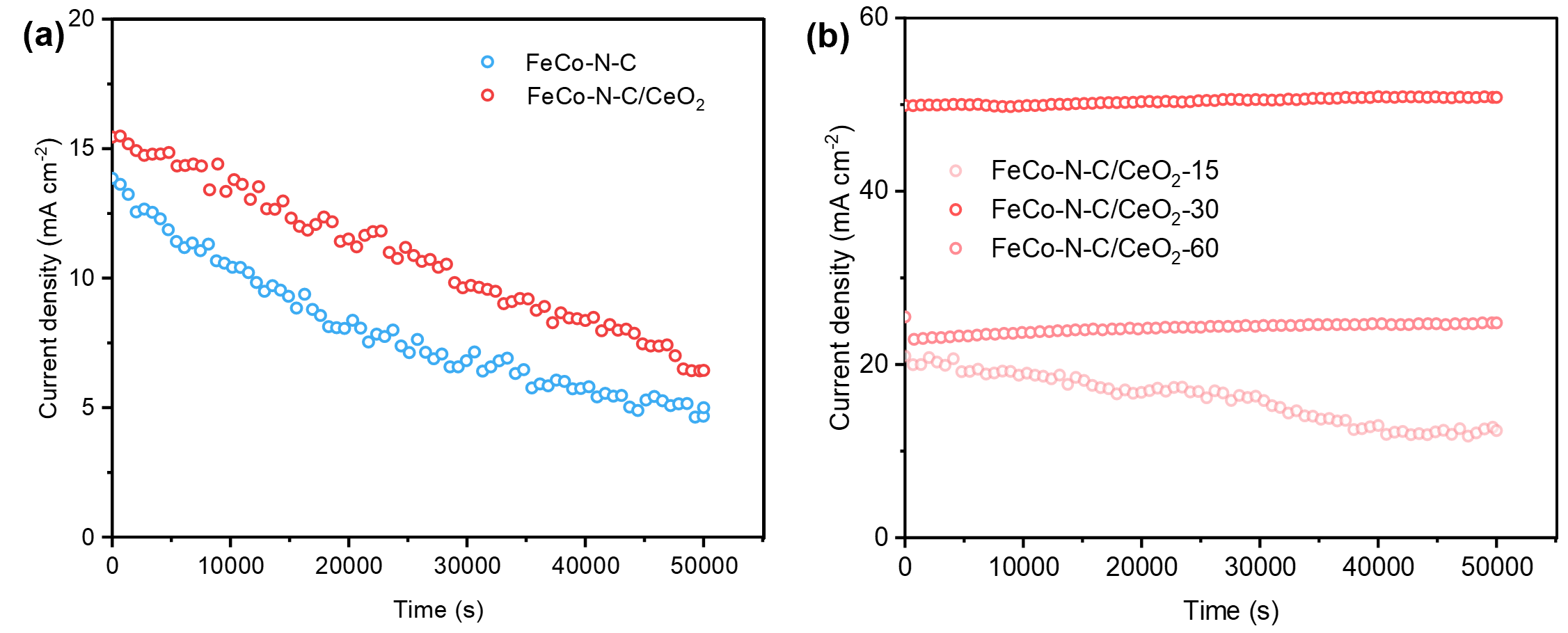


**Figure S25.** The i-t curves of a) FeCo–N–C, FeCo–N–C/CeO_2_, b) FeCo–N–C/CeO_2_-15, FeCo–N–C/CeO_2_-30, and FeCo–N–C/CeO_2_-60 recorded at 1.58 V vs. RHE.


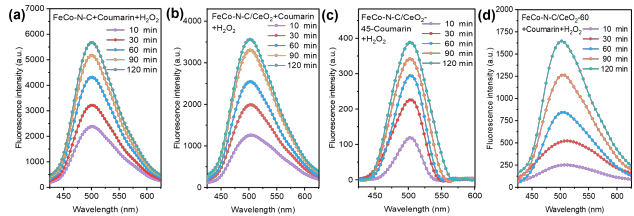


**Figure S26.** Fluorescence spectra of coumarin dye after reaction with radicals generated by the Fenton reaction system composed of a) FeCo–N–C, b) FeCo–N–C/CeO_2_, c) FeCo–N–C/CeO_2_-45 and d) FeCo–N–C/CeO_2_-60 with H_2_O_2_ at reaction times of 10, 30, 60, 90, and 120 min.


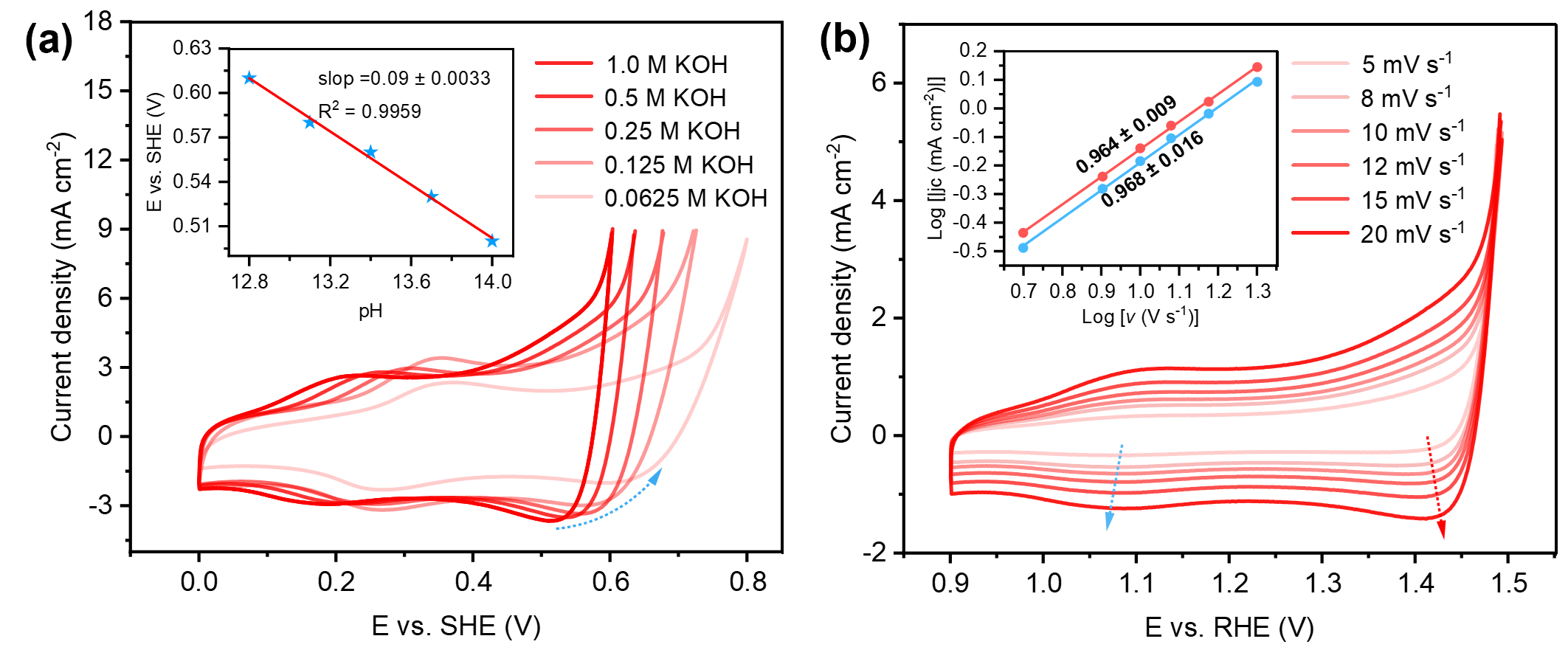


**Figure S27.** a) The CV curves of the FeCo–N–C recorded in KOH solutions of different pH values, with the inset showing the relationship between the reduction peak potential (vs. SHE) and the solution pH. b) The magnified CV curves of FeCo–N–C at different scan rates in 1.0 M KOH solution, with the inset showing the logarithmic relationship between the current density *j*_c_ and the scan rate *v*.


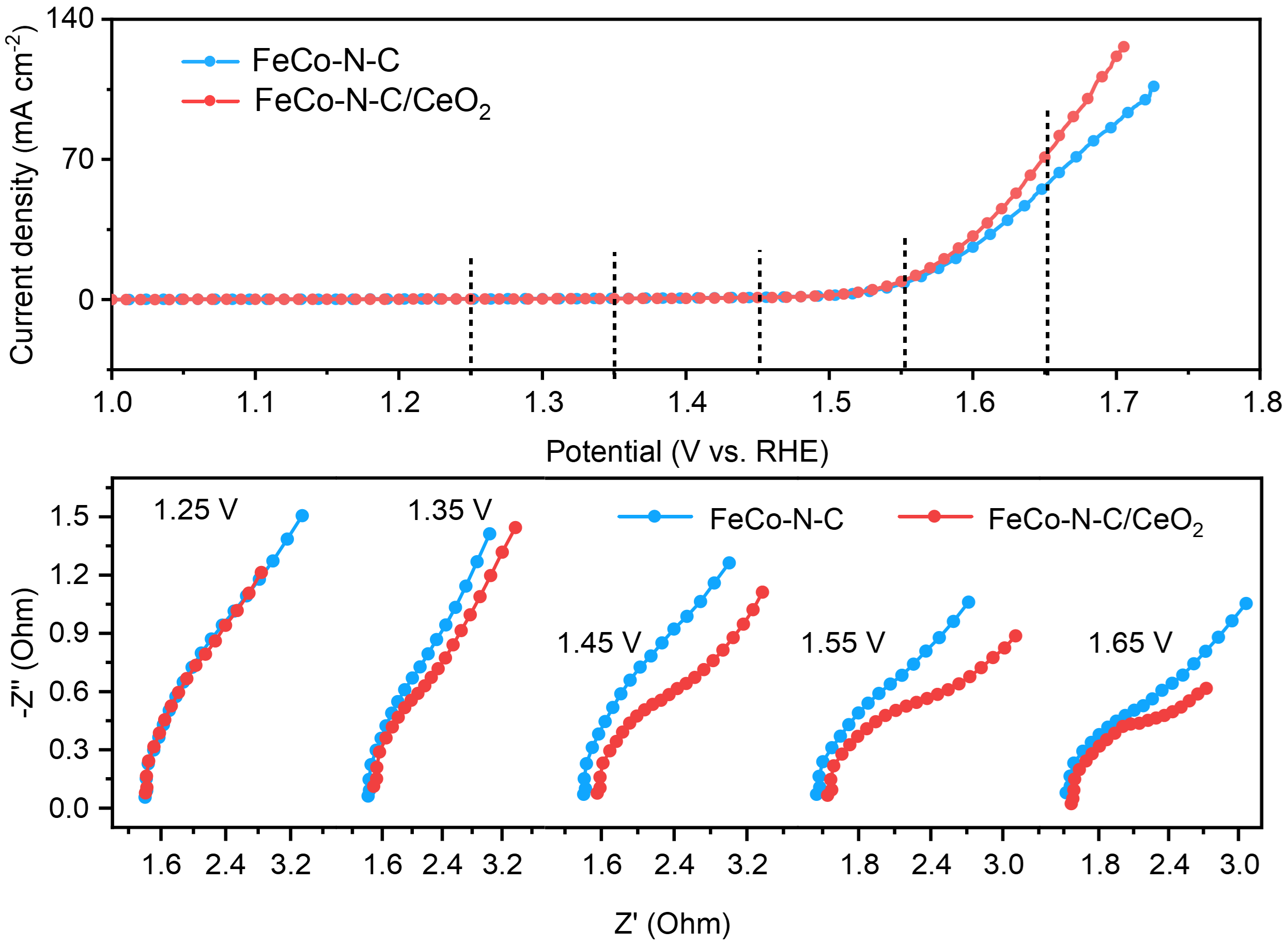


**Figure S28.** Nyquist plots for FeCo–N–C and FeCo–N–C/CeO_2_ during the OER process. The change of charge-transfer resistance (R_ct_) is indicated by a smaller radius of the semicircle at higher applied potentials.


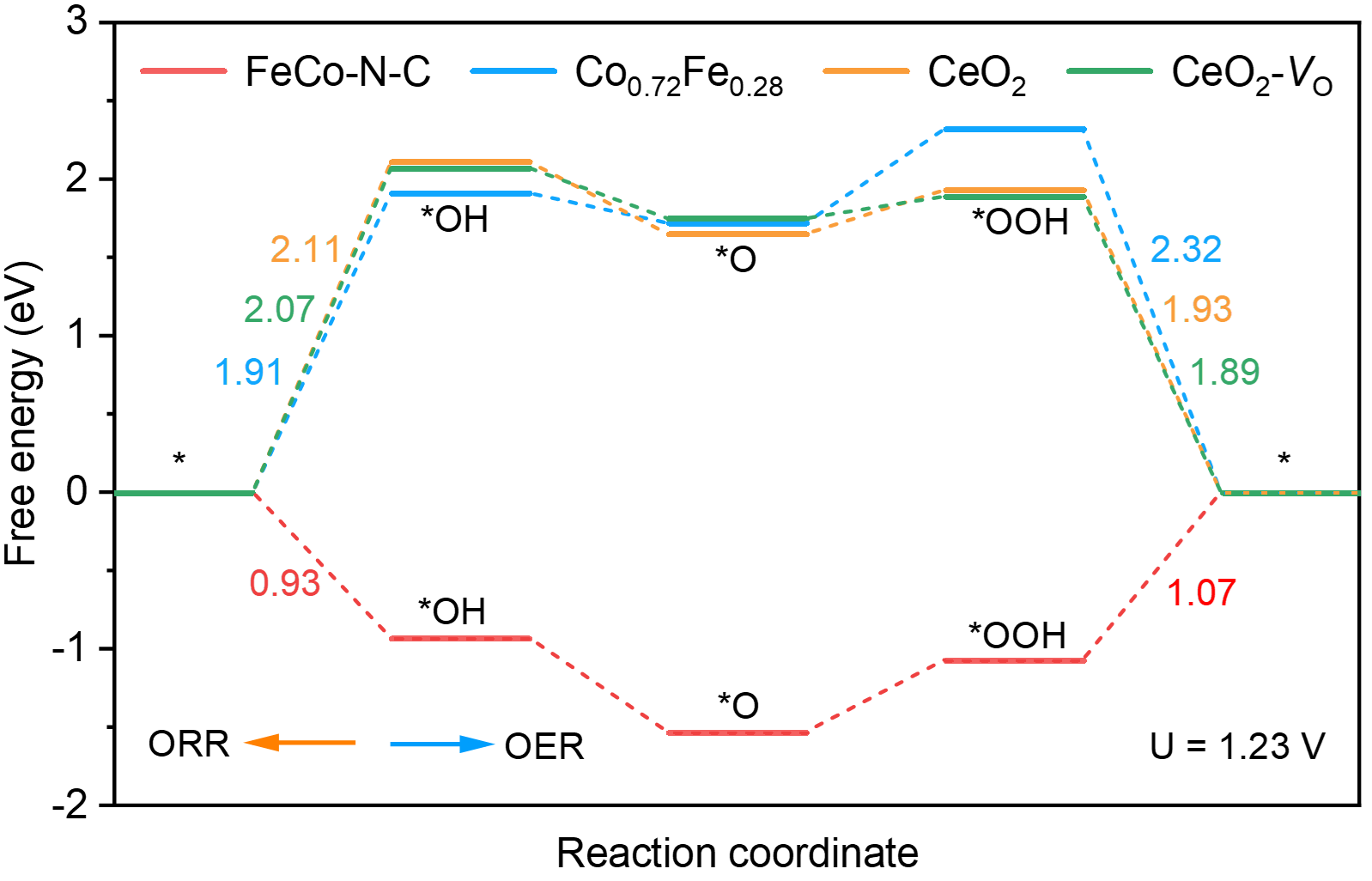


**Figure S29.** Free energies of the intermediates for the OER (left to right) and ORR (right to left) in the FeCo–N–C, Co_0.72_Fe_0.28_, CeO_2_ and CeO_2_-*V*_O_ catalysts.


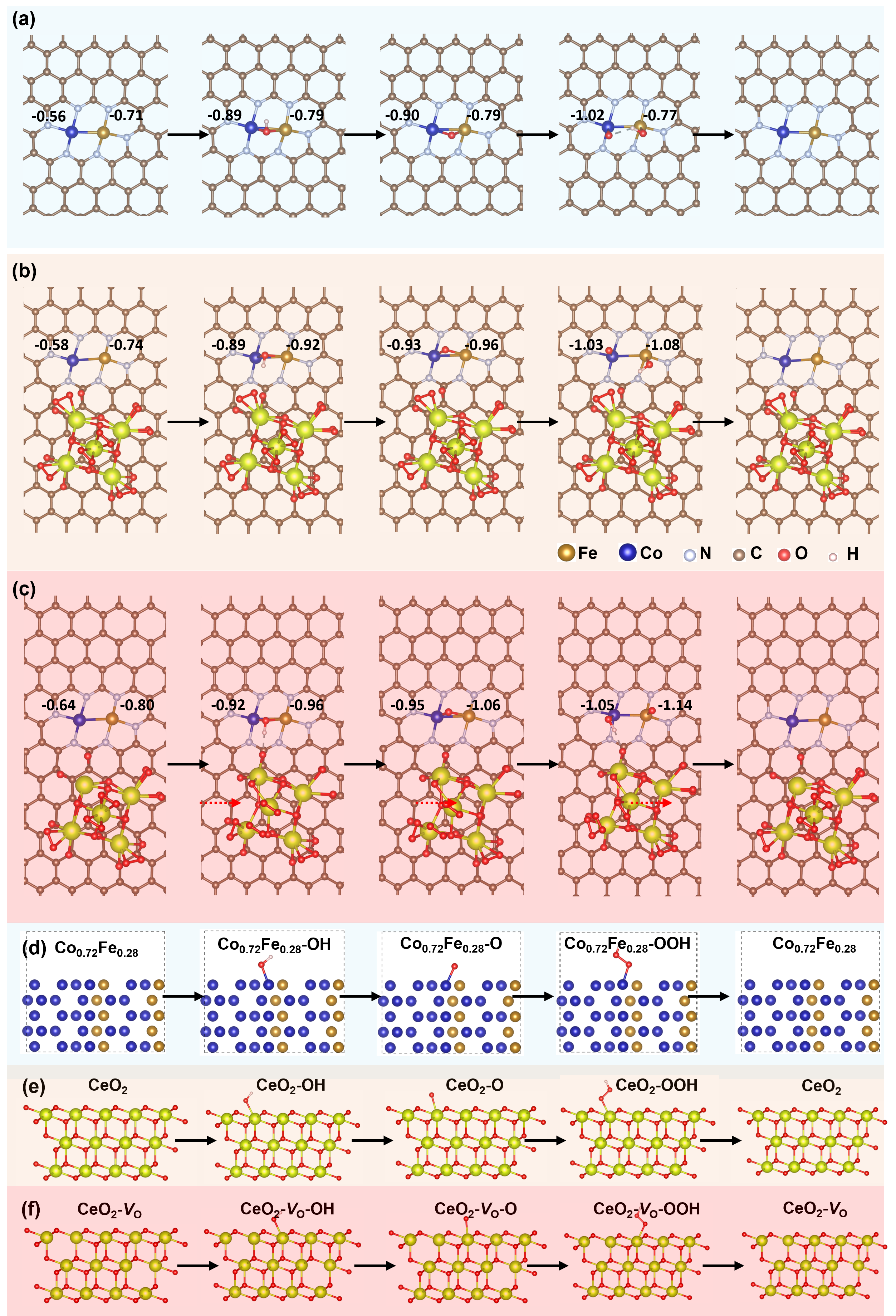


**Figure S30.** The OER reaction processes of a) FeCo–N–C, b) FeCo–N–C/CeO_2_, and c) FeCo–N–C/CeO_2_-45, d) Co_0.72_Fe_0.28_, e) CeO_2_ and f) CeO_2_-*V*_O_, along with the Bader charges of Fe and Co sites at each reaction step.


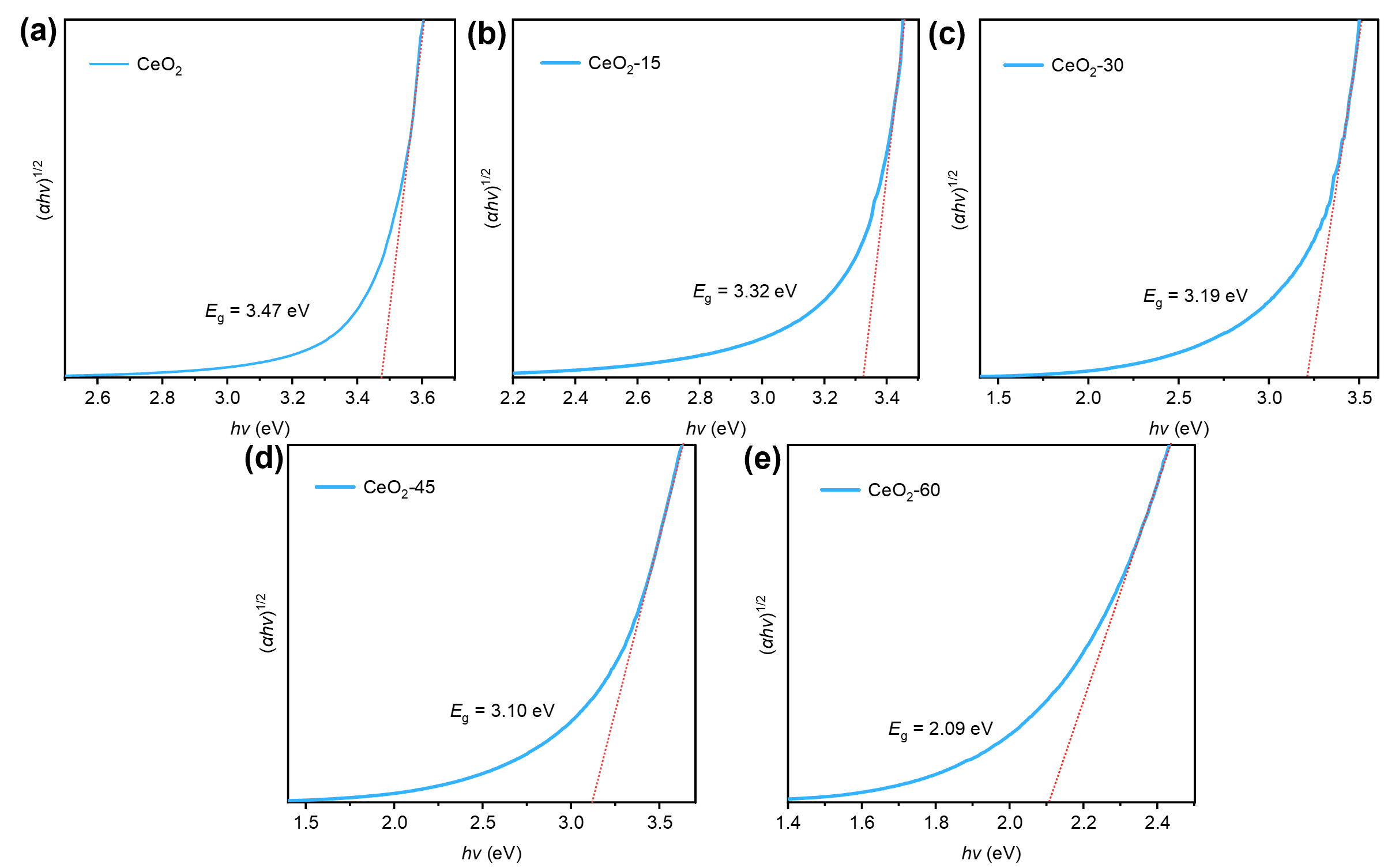


**Figure S31.** Band gap spectra of a) CeO_2_, b) CeO_2_-15, c) CeO_2_-30, d) CeO_2_-45, and e) CeO_2_-60.


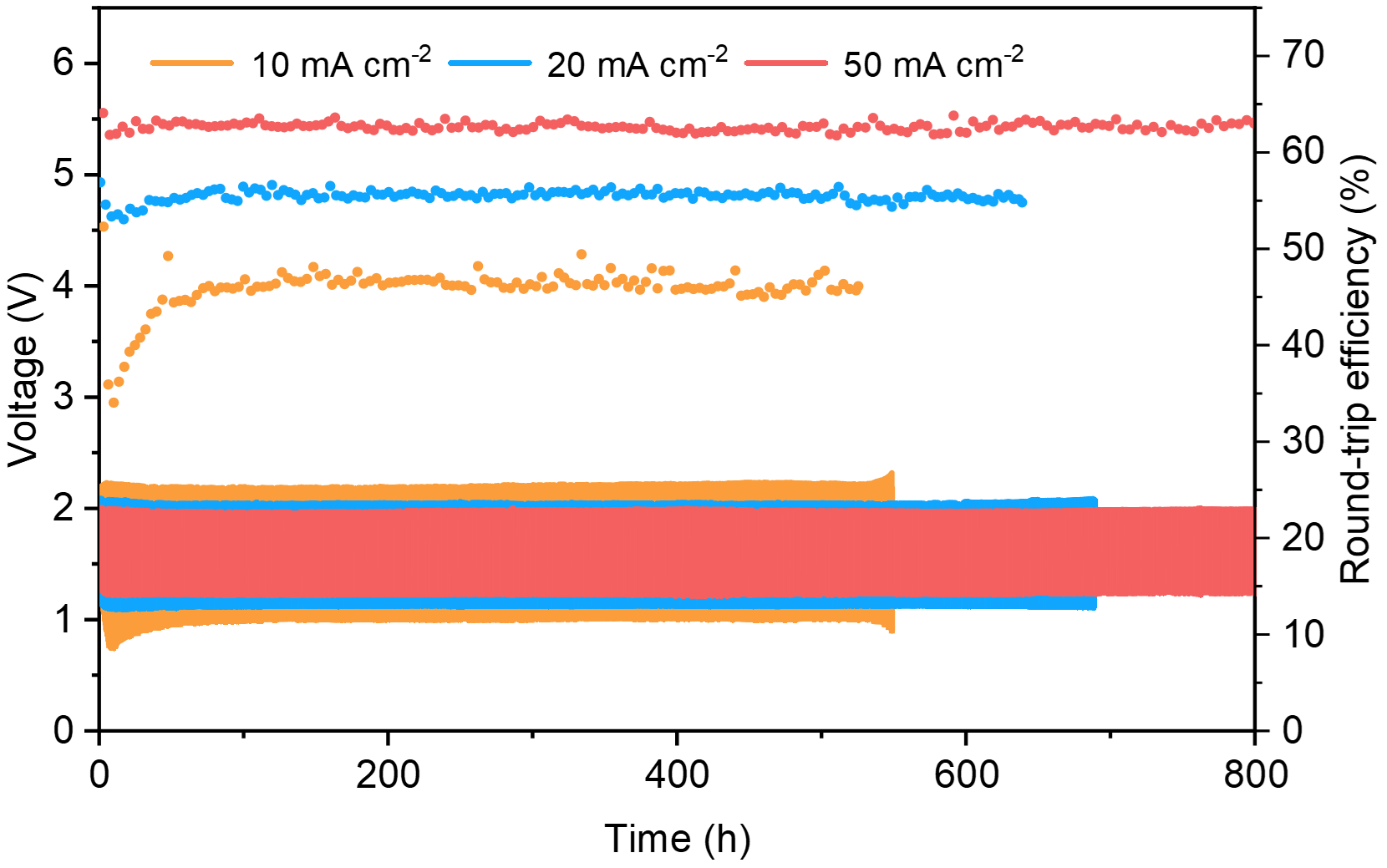


**Figure S32.** Galvanostatic charge/discharge cycling curves of the FeCo–N–C/CeO_2_-45 catalyst at 10 mA cm^-2^ and the corresponding round-trip efficiency.


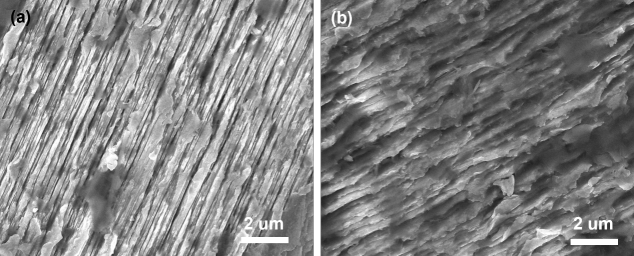


**Figure S33.** SEM images of the zinc anode a) before and b) after cycling in the zinc–air battery.


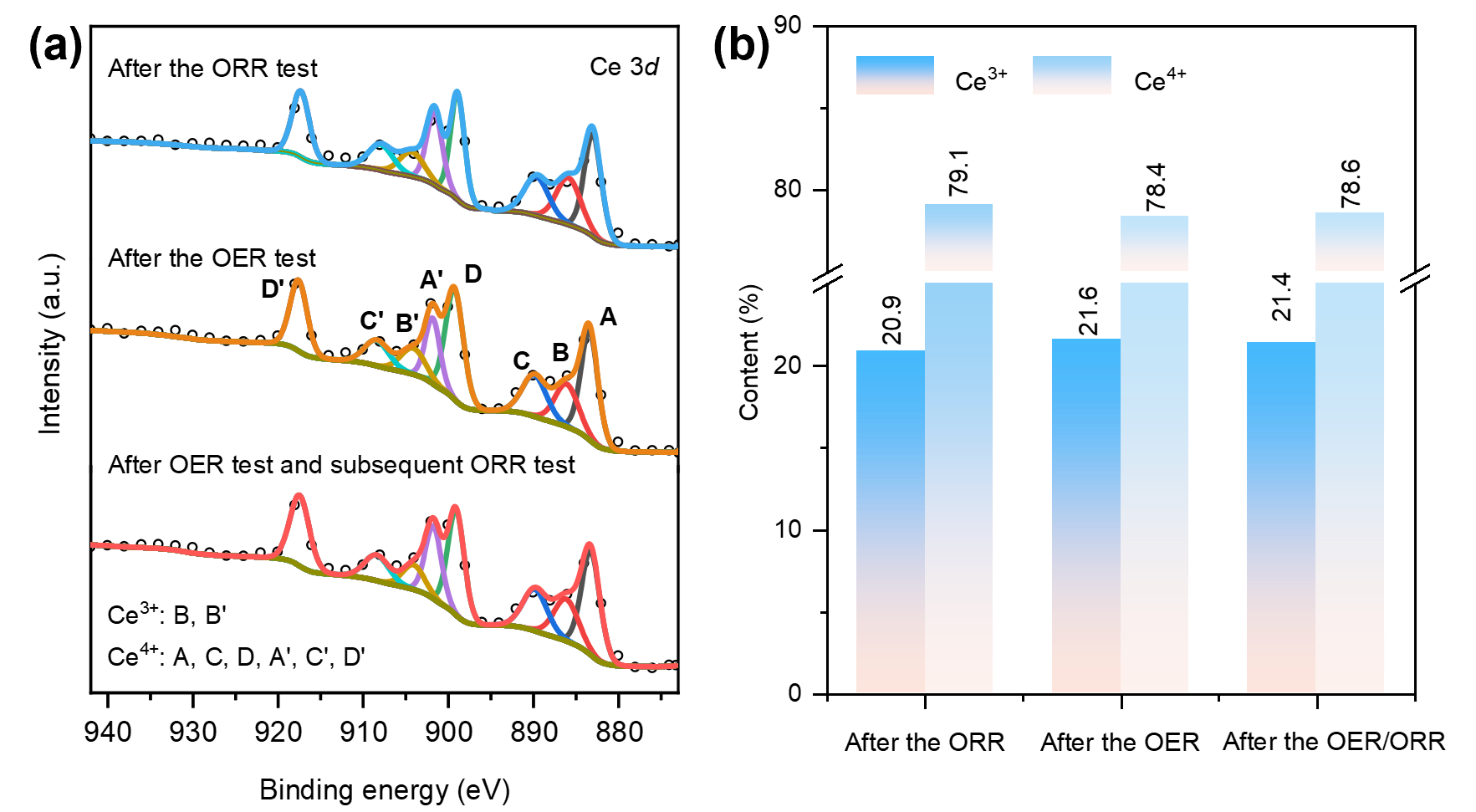


**Figure S34.** a) The Ce 3*d* spectra of FeCo–N–C/CeO_2_-45 after electrochemical testing and b) the variation trends of Ce^3+^ and Ce^4+^ after electrochemical testing. For ORR, FeCo–N–C/CeO_2_-45 catalyst underwent 5,000 CV cycles at a scan rate of 20 mV s^-1^ within the potential range of 0.6–0.9 V. For OER, a chronoamperometric test was conducted on the FeCo–N–C/CeO_2_-45 catalyst at a potential of 1.58 V for 50,000 s. For ZABs, the FeCo-N-C/CeO_2_-45 catalyst was assembled into a ZABs and subjected to charge-discharge cycling (OER first, followed by ORR) for 100 h at a current density of 10 mA cm^-2^.

**Table S1.** The percentages (%) of different N species in the samples obtained by XPS analysis

| **Samples** | **pyridine nitrogen** | **pyrrole nitrogen** | **graphitic nitrogen** | **oxidized nitrogen** |
| --- | --- | --- | --- | --- |
| **FeCo–N–C** | 36.09 | 20.99 | 22.82 | 21.50 |
| **FeCo–N–C/CeO_2_** | 36.35 | 20.74 | 22.97 | 19.94 |
| **FeCo–N–C/CeO_2_-15** | 36.47 | 19.98 | 23.12 | 20.43 |
| **FeCo–N–C/CeO_2_-30** | 37.83 | 19.77 | 23.84 | 18.56 |
| **FeCo–N–C/CeO_2_-45** | 38.40 | 19.00 | 24.01 | 18.58 |
| **FeCo–N–C/CeO_2_-60** | 37.03 | 20.13 | 23.96 | 18.89 |

**Table S2.** Comparison of the ZABs performance of FeCo–N–C/CeO_2_-45 to another related bifunctional catalysts in literature^1-15^

| **Catalysts** | **E_1/2_ (V vs. RHE)** | **η @ 10 mA cm^-2^ (V)** | **△E (V)** | **Power density**  **(mW cm^-2^)** | **Specific capacity**  **(mAh g^-1^)** | **Cycling stability (h)** | **Ref.** |
| --- | --- | --- | --- | --- | --- | --- | --- |
| Co_6_Mo_6_C_2_/HNC | 0.865 | 0.284 | 0.649 | 224.8 | 794.83 | 67 | 1 |
| Co@H-NCNT | 0.851 | 0.351 | 0.730 | 207.0 | 879.70 | 100 | 2 |
| Fe-Se/NC | 0.925 | 0.393 | 0.698 | 135.0 | 764.00 | 200 | 3 |
| Pt=N_2_=Fe ABA | 0.950 | - | - | 198.4 | 787.80 | 30 | 4 |
| Co-N-CTS | 0.840 | 0.290 | 0.680 | 140.0 | - | 600 | 5 |
| FeCo NC/D | 0.892 | 0.362 | 0.700 | 157.0 | 725.00 | 40 | 6 |
| CoFe-NCNFs | 0.850 | 0.323 | 0.703 | 116.1 | - | 110 | 7 |
| 3CS_0.05_-PBCC | - | 0.300 | - | - | - | 150 | 8 |
| O-Co-N/C | 0.850 | 0.330 | 0.710 | 143.0 | 785.00 | 150 | 9 |
| MnO@CNT@Co-N/C | 0.810 | 0.390 | 0.810 | 200.8 | 802.70 | 300 | 10 |
| CNT@SAC-Co/NCP | 0.870 | 0.380 | 0.740 | 172.0 | 864.00 | 37 | 11 |
| (imidazolyl)porphyrin@ZIF-67 | 0.790 | 0.416 | 0.856 | 220.0 | 654.00 | 110 | 12 |
| NiCo_2_O_4_ | 0.760 | 0.313 | 0.783 | 71.0 | - | 79 | 13 |
| Fe SAS/NC | 0.850 | 0.320 | 0.700 | 306.1 | 785.40 | 315 | 14 |
| FeMn-DSAC | 0.922 | 0.405 | 0.713 | 184.0 | 734.00 | 80 | 15 |
| Pt/C + RuO_2_ | 0.845 | 0.319 | 0.704 | 107.0 | 686.90 | 43 | This work |
| FeCo–N–C/CeO_2_-45 | 0.890 | 0.285 | 0.625 | 335.0 | 801.00 | 1000 |  |

**3.References in Supporting Information**

(1) Fan, F.; Hui, Y.; Devasenathipathy, R.; Peng, X.; Huang, Q.; Xu, W.; Yang, F.; Liu, X.; Wang, L.; Chen, D.-H.; et al. Composition-adjustable Mo_6_Co_6_C_2_/Co@carbon nanocage for enhanced oxygen reduction and evolution reactions. *J. Colloid Interface Sci.* **2023**, *636*, 450-458.

(2) Choi, E. Y.; Kim, D. E.; Lee, S. Y.; Park, C. B.; Kim, C. K. Cobalt nanoparticles-encapsulated holey nitrogen-doped carbon nanotubes for stable and efficient oxygen reduction and evolution reactions in rechargeable Zn-air batteries. *Appl. Catal. B: Environ.* **2023**, *325*, 122386.

(3) Wang, Y.; Wu, J.; Tang, S.; Yang, J.; Ye, C.; Chen, J.; Lei, Y.; Wang, D. Synergistic Fe-Se Atom Pairs as Bifunctional Oxygen Electrocatalysts Boost Low-Temperature Rechargeable Zn-Air Battery. *Angew. Chem. Int. Ed.* **2023**, *62* (15), e202219191.

(4) Zhou, W.; Su, H.; Cheng, W.; Li, Y.; Jiang, J.; Liu, M.; Yu, F.; Wang, W.; Wei, S.; Liu, Q. Regulating the scaling relationship for high catalytic kinetics and selectivity of the oxygen reduction reaction. *Nat. Commun.* **2022**, *13* (1), 6414.

(5) Shi, W.; Li, Z.; Gong, Z.; Liang, Z.; Liu, H.; Han, Y.-C.; Niu, H.; Song, B.; Chi, X.; Zhou, J. Transient and general synthesis of high-density and ultrasmall nanoparticles on two-dimensional porous carbon via coordinated carbothermal shock. *Nat. Commun.* **2023**, *14*, 2294.

(6) Kim, K.; Min, K.; Go, Y.; Lee, Y.; Shim, S. E.; Lim, D.; Baeck, S.-H. FeCo alloy nanoparticles embedded in N-doped carbon supported on highly defective ketjenblack as effective bifunctional electrocatalysts for rechargeable Zn-air batteries. *Appl. Catal. B: Environ.* **2022**, *315*, 121501.

(7) Lin, S.-Y.; Chen, Y.-P.; Cao, Y.; Zhang, L.; Feng, J.-J.; Wang, A.-J. Aminouracil-assisted synthesis of CoFe decorated bougainvillea-like N-doped carbon nanoflowers for boosting Zn-air battery and water electrolysis. *J. Power Sources* **2022**, *521*, 230926.

(8) Wang, Y.; Ge, X.; Lu, Q.; Bai, W.; Ye, C.; Shao, Z.; Bu, Y. Accelerated deprotonation with a hydroxy-silicon alkali solid for rechargeable zinc-air batteries. *Nat. Commun.* **2023**, *14*, 6968.

(9) Zhang, W.; Xu, C. H.; Zheng, H.; Li, R.; Zhou, K. Oxygen‐Rich Cobalt-Nitrogen-Carbon Porous Nanosheets for Bifunctional Oxygen Electrocatalysis. *Adv. Funct. Mater.* **2022**, *32*, 2200763.

(10) Li, F.; Qin, T.; Sun, Y.; Jiang, R.; Yuan, J.; Liu, X.; O'Mullane, A. P. Preparation of a one-dimensional hierarchical MnO@CNT@Co-N/C ternary nanostructure as a high-performance bifunctional electrocatalyst for rechargeable Zn–air batteries. *J. Mater. Chem. A* **2021**, *9*, 22533-22543.

(11) Li, J. C.; Meng, Y.; Zhang, L.; Li, G.; Shi, Z.; Hou, P. X.; Liu, C.; Cheng, H. M.; Shao, M. Dual‐Phasic Carbon with Co Single Atoms and Nanoparticles as a Bifunctional Oxygen Electrocatalyst for Rechargeable Zn–Air Batteries. *Adv. Funct. Mater.* **2021**, *31*, 2103360.

(12) Liang, Z.; Guo, H.; Zhou, G.; Guo, K.; Wang, B.; Lei, H.; Zhang, W.; Zheng, H.; Apfel, U. P.; Cao, R. Metal–Organic‐Framework‐Supported Molecular Electrocatalysis for the Oxygen Reduction Reaction. *Angew. Chem. Int. Ed.* **2021**, *60*, 8472-8476.

(13) Li, H.; Wang, J.; Tjardts, T.; Barg, I.; Qiu, H.; Müller, M.; Krahmer, J.; Askari, S.; Veziroglu, S.; Aktas, C.; et al. Plasma‐Engineering of Oxygen Vacancies on NiCo_2_O_4_ Nanowires with Enhanced Bifunctional Electrocatalytic Performance for Rechargeable Zinc‐air Battery. *Small* **2024**, *20*, 2310660.

(14) Li, Z.; Ji, S.; Xu, C.; Leng, L.; Liu, H.; Horton, J. H.; Du, L.; Gao, J.; He, C.; Qi, X.; et al. Engineering the Electronic Structure of Single-Atom Iron Sites with Boosted Oxygen Bifunctional Activity for Zinc-Air Batteries. *Adv. Mater.* **2022**, *35*, 2209644.

(15) Cui, T.; Wang, Y. P.; Ye, T.; Wu, J.; Chen, Z.; Li, J.; Lei, Y.; Wang, D.; Li, Y. Engineering Dual Single‐Atom Sites on 2D Ultrathin N‐doped Carbon Nanosheets Attaining Ultra-Low-Temperature Zinc-Air Battery. *Angew. Chem. Int. Ed.* **2022**, *61* (12), e202115219.
